# Unlocking Li superionic conductivity in face-centred cubic oxides via face-sharing configurations

**DOI:** 10.1038/s41563-024-01800-8

**Published:** 2024-02-02

**Authors:** Yu Chen, Zhengyan Lun, Xinye Zhao, Krishna Prasad Koirala, Linze Li, Yingzhi Sun, Christopher A. O’Keefe, Xiaochen Yang, Zijian Cai, Chongmin Wang, Huiwen Ji, Clare P. Grey, Bin Ouyang, Gerbrand Ceder

**Affiliations:** 1 Department of Materials Science and Engineering, University of California, Berkeley, CA USA; 2 Materials Sciences Division, Lawrence Berkeley National Laboratory, Berkeley, CA USA; 3 Yusuf Hamied Department of Chemistry, University of Cambridge, Cambridge, UK; 4 School of Chemical Sciences, University of Chinese Academy of Sciences, Beijing, China; 5 Physical and Computational Sciences Directorate, Pacific Northwest National Laboratory, Richland, WA USA; 6 Environmental Molecular Sciences Laboratory, Pacific Northwest National Laboratory, Richland, WA USA; 7 Department of Materials Science and Engineering, University of Utah, Salt Lake City, UT USA; 8 Department of Chemistry and Biochemistry, Florida State University, Tallahassee, FL USA

**Keywords:** Batteries, Ceramics, Solid-state chemistry

## Abstract

Oxides with a face-centred cubic (fcc) anion sublattice are generally not considered as solid-state electrolytes as the structural framework is thought to be unfavourable for lithium (Li) superionic conduction. Here we demonstrate Li superionic conductivity in fcc-type oxides in which face-sharing Li configurations have been created through cation over-stoichiometry in rocksalt-type lattices via excess Li. We find that the face-sharing Li configurations create a novel spinel with unconventional stoichiometry and raise the energy of Li, thereby promoting fast Li-ion conduction. The over-stoichiometric Li–In–Sn–O compound exhibits a total Li superionic conductivity of 3.38 × 10^−4^ S cm^−1^ at room temperature with a low migration barrier of 255 meV. Our work unlocks the potential of designing Li superionic conductors in a prototypical structural framework with vast chemical flexibility, providing fertile ground for discovering new solid-state electrolytes.

## Abstract (web summary)

Oxides with a face-centred cubic anion sublattice are generally not considered as solid-state electrolytes. Li superionic conductivity in face-centred cubic oxides with face-sharing Li configurations have now been created through cation over-stoichiometry in rocksalt-type lattices via excess Li.

## Main

**Fig. 1:**
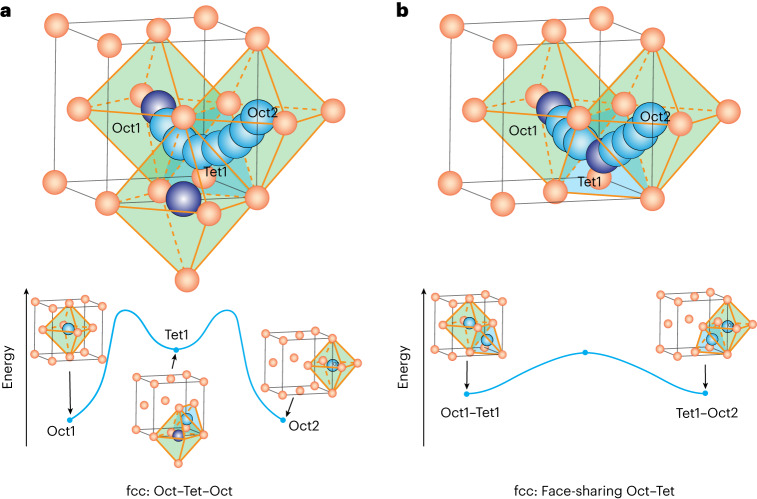
Schematic illustration of Li-ion migration pathways in fcc-type oxides. **a**, Oct–Tet–Oct pathway in fcc oxygen lattice without face-sharing Li in the initial state (top) and the energy landscape (bottom). **b**, Oct–Tet/Tet–Oct pathways in fcc oxygen lattice with face-sharing Li in the initial state (top) and the energy landscape (bottom). Orange, oxygen anions; purple, Li ions in the initial sites; blue, Li ions in the migration paths.

The tremendous interest in all-solid-state batteries (ASSBs), driven by their enhanced safety and potential compatibility with a lithium (Li) metal anode, has brought solid-state superionic conductors (SICs) to the forefront of battery research^1,2^. SICs with high ionic conductivities as well as excellent chemical and electrochemical stability are the key to realizing practical ASSBs^3,4^. So far, multiple sulfide-based SICs, including Li_10_GeP_2_S_12_ (ref. ^5^), Li_9.54_Si_1.74_P_1.44_S_11.7_Cl_0.3_ (ref. ^6^) and Li argyrodites^7^, have been found to have high Li-ion conductivity, exceeding that of conventional liquid electrolytes (~10 mS cm^−1^). However, the narrow electrochemical stability window, poor chemical stability and high moisture sensitivity of sulfides have limited their application in ASSBs^8–11^. Oxide-based SICs tend to have better air and electrochemical stability, but at the cost of lower ionic conductivity^3,12^. Thus far, room-temperature (RT) Li-ion conductivity values of >0.1 mS cm^−1^ have been reported only in a handful of oxides (for example, Li garnets^13,14^, NASICON-type Li oxides^15^, and Li perovskites^16^), all of which have uncommon oxygen packing. Surprisingly, the most common class of oxides, those with a close-packed face-centred cubic (fcc) anion sublattice, have so far not displayed high enough Li-ion mobility to be considered as SICs. The traditional rationale for excluding fcc-type oxides from the search for potential SICs is that the activation energy for Li motion is expected to be high, because the Li-ion migration path must cross through both tetrahedral (Tet) and octahedral (Oct) sites with very different site energies^17^, as shown in Fig. 1a.

As a strategy to enhance the Li-ion conductivity in fcc-type oxides, we propose to construct a face-sharing Li configuration in which Li ions simultaneously occupy the neighbouring sites that are face-sharing. As shown in Fig. 1b, such a face-sharing configuration raises the energy of the Li ion and thus flattens the energy landscape for ion migration. Furthermore, the strong Li–Li interactions between face-sharing sites can activate concerted ion migration, as reported for many SICs^18,19^. The formation of distorted face-sharing Li–O polyhedra has also emerged as a key mechanism to account for the fast kinetics observed in several Li-ion anodes (for example, Li_4_Ti_5_O_12_ (ref. ^20^) and Li_3_V_2_O_5_ (ref. ^21^)).

In this work we demonstrate that we can create face-sharing configurations in fcc-type oxides by incorporating excess Li to yield cation over-stoichiometry (Li over-stoichiometry for short) in a Li–In–Sn–O compound (o-LISO). The resulting material exhibits a Li-ion conductivity of 3.38 × 10^−4^ S cm^−1^ at RT with a remarkably low migration barrier of 255 meV, which is notably superior to rocksalts that contain a stoichiometric amount of cations. Furthermore, we observe a surprising spinel-like ordering of the cations even though the cation/anion ratio is >1 in this compound. This phenomenon leads to a unique nanosized phase (s-phase) with improved three-dimensional (3D) percolation of face-sharing Li and enhanced ionic conductivity compared with that of the cation-disordered state. This success motivated us to further investigate the thermodynamic stability of over-stoichiometric rocksalt-type oxides composed of many other metal species, with the aim of providing guidelines to accelerate future SIC exploration within this vast new design space.

## Li superionic conductivity in o-LISO

Stoichiometric rocksalt-type oxides feature a cation-to-anion ratio of 1. To create a face-sharing Li configuration, we introduce excess Li to form an over-stoichiometric rocksalt-type (ORX) oxide. As an example, Li_17_In_9_SnO_24_ (o-LISO), was synthesized using a conventional solid-state method. The synchrotron X-ray diffraction pattern of o-LISO (Supplementary Fig. 1) shows sharp peaks of a rocksalt lattice along with additional broad peaks, which together can be indexed to the $Fd\bar{3}m$ space group. A detailed structure analysis is presented in the subsequent sections.

**Fig. 2:**
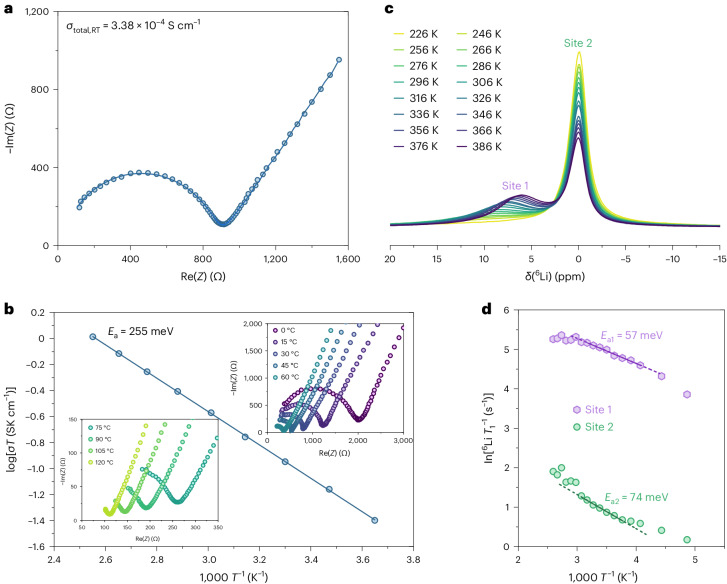
Li-ion conductivity of o-LISO. **a**, Nyquist plot from EIS measurements at RT and the corresponding fit to the equivalent circuit. **b**, Insets: Nyquist plots in the temperature range from 0 to 120 °C. Arrhenius plot of the total ionic conductivity. **c**, ^6^Li magic-angle spinning (MAS) ssNMR spectra of o-LISO at temperatures from 226 to 386 K. **d**, Temperature dependence of the ^6^Li ssNMR spin–lattice relaxation rates of the two Li sites in o-LISO. Source data

The Li-ion conductivity of o-LISO was measured using electrochemical impedance spectroscopy (EIS). As shown in Fig. 2a,b, the Nyquist plots consist of a semicircle at high frequency and a linear tail at low frequency, characteristic of purely ionic conductors. The equivalent-circuit-fitted total ionic conductivity (*σ*_total_) of o-LISO, including both the bulk and grain-boundary contributions, is 3.38 × 10^−4^ S cm^−1^ at RT (Supplementary Fig. 2 and Note 1). The activation energy (*E*_a_) of Li-ion conduction in o-LISO is estimated to be 255 meV from a linear fit to the Arrhenius plot (Fig. 2b). A direct-current polarization experiment gives an RT electronic conductivity of 2.47 × 10^−9^ S cm^−1^ (Supplementary Fig. 3), five orders of magnitude lower than the ionic conductivity. This performance of o-LISO is comparable to that of current state-of-the-art oxide-based SICs (for example, garnet-type Li_7_La_3_Zr_2_O_12_ (ref. ^14^) and NASICON-type Li_1.3_Al_0.3_Ti_1.7_(PO_4_)_3_ (ref. ^15^)) and is dramatically superior to that of typical fcc-type Li–metal oxides reported in the literature, for example, Li_2_MgTiO_4_ (~9 × 10^−5^ S cm^−1^ at 600 °C, *E*_a_ = 0.53 eV)^22^, Li_2.8_Ni_0.1_NbO_4_ (~5 × 10^−4^ S cm^−1^ at 300 °C, *E*_a_ ≈ 0.6 eV)^23^ and Li_4_Ti_5_O_12_ (~7 × 10^−8^ S cm^−1^ at RT, *E*_a_ ≈ 0.5 eV)^24^. Notably, the activation energy is lower than that of most oxide SICs (Supplementary Fig. 4), indicating that facile Li-ion conduction is achieved in this ORX compound.

The Li-ion dynamics in o-LISO were probed further using variable-temperature ^6^Li solid-state NMR (ssNMR) from 226 to 386 K. The spectra in Fig. 2c can be fitted with two Li sites, which are assigned to Tet (site 1, higher chemical shift *δ*) and Oct (site 2, lower chemical shift) coordination environments, respectively^25^. With increasing temperature *T*, a narrowing of the peak width for both sites is observed, where this is much more pronounced for site 1, dropping from a full-width at half-height of more than 10 ppm to less than 7 ppm. This narrowing (coupled with the extremely short relaxation times, Supplementary Table 1) suggests enhanced Li mobility involving this site. In addition, the integrated intensity ratio between site 1 and site 2 increases as the temperature rises from approximately 1:4 at 226 K and approaches 1:1 at 386 K (Supplementary Table 1), which implies a thermal population of a higher energy site at higher temperatures, presumably enabled by Li hopping. The local Li-hopping barriers are 57 meV for site 1 and 74 meV for site 2 from fitting the Arrhenius plot of the ^6^Li spin–lattice relaxation rate (*T*_1_^−^^1^) with respect to the inverse temperature (Fig. 2d). These values are much smaller than the apparent activation energy determined from EIS (that is, 255 meV) because EIS measures the overall macroscopic Li-ion conductivity that includes the contributions from temperature-dependent correlated motion, grain boundaries and pores, whereas NMR probes the rate of local ion hopping^26^. The NMR-probed Li-hopping barriers of o-LISO are much smaller than those of other SICs reported in the literature^27–29^, suggesting a very high intrinsic Li mobility in the structure.

## Effect of over-stoichiometric Li on phase formation

**Fig. 3:**
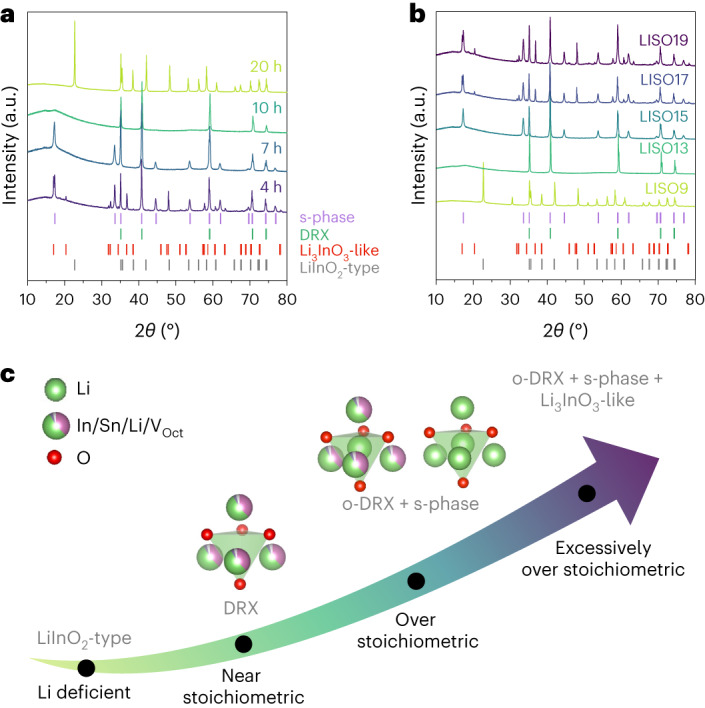
LISO phase evolution with varying Li content and calcination time. **a**, X-ray diffraction patterns of LISO samples calcined at 1,050 °C for 4 h, 7 h, 10 h and 20 h to achieve an increasing degree of Li evaporation. All start from the same nominal composition of Li_17_In_9_SnO_24_. The Bragg positions of the phases identified are labelled by vertical tick marks: purple, s-phase; green, DRX; red, Li_3_InO_3_-like phase; and grey, LiInO_2_-type phase. **b**, X-ray diffraction patterns of LISO*n* (*n* = 9, 13, 15, 17 and 19) calcined at 1,050 °C for 4 h. The vertical tick marks denote the same as those in **a**. **c**, Schematic of phase evolution with increasing Li content in the LISO system. o-DRX refers to the DRX phase with an over-stoichiometric Li content. V_Oct_, octahedral vacancy. Source data

To understand the observed Li superionic conductivity in o-LISO, we investigated the phase evolution as function of the amount of over-stoichiometry. Figure 3a presents the X-ray diffraction patterns of the LISO samples prepared using the same precursors targeting the composition Li_17_In_9_SnO_24_ but with different calcination times. When calcinated at 1,050 °C for 4 h, three phases can be identified in the X-ray diffraction pattern: cation-disordered rocksalt (DRX), an unknown phase with spinel-like reflections (denoted as the ‘s-phase’) and a Li_3_InO_3_-like phase^30^ (see Supplementary Fig. 5 and Note 2 for details of the structure analysis). With an increasing calcination time, the Li_3_InO_3_-like phase disappears first, followed by the s-phase, leaving a pure DRX phase after calcinating for 10 h. Extending the calcination time further to 20 h eventually results in a sole LiInO_2_-type phase present. Thermogravimetric analysis showed a continuous mass loss in the material when held at 1,050 °C (Supplementary Fig. 6), which must arise from Li_2_O loss given its volatile nature and the highly Li-rich starting composition. We thus speculate that the observed phase evolution is driven by a decreasing Li content with prolonged annealing. To confirm this speculation, a series of compositions of Li_*n*_In_9_SnO_(31+*n*)/2_ (LISO*n*, *n* = 9, 13, 15, 17 and 19) with the same In/Sn ratio but various Li contents were calcinated at 1,050 °C for 4 h. The resulting X-ray diffraction patterns are shown in Fig. 3b. With increasing Li content, the phases formed change from LiInO_2_-type, to DRX, to a mixed DRX and s-phase, and finally to a mixture with the Li_3_InO_3_-like phase. This trend, summarized in Fig. 3c, is consistent with the phases observed in Fig. 3a, if indeed the Li content decreases with increasing calcination time.

Furthermore, even though both o-LISO and near-stoichiometric LISO (ns-LISO; synthesized via a longer calcination time, see Methods for more details) contain a DRX phase, the diffraction peaks of the former appear at a lower Bragg position (2*θ*) than for the latter (Supplementary Fig. 7). Such lattice expansion of the DRX phase in o-LISO probably stems from the additional Li incorporated into Tet sites. We therefore differentiate the DRX phase in o-LISO by denoting it as ‘o-DRX’. In addition, we observe the formation of a unique s-phase along with o-DRX in o-LISO, which is rather counterintuitive because oxide spinels typically form for cation-deficient compositions. The structure of this s-phase will be discussed in the next section.

Elemental analysis performed using inductively coupled plasma optical emission spectroscopy confirmed that the Li content in o-LISO (Li:In:Sn = 14.9:9.0:1.0) is indeed over-stoichiometric and much higher than that in ns-LISO (Li:In:Sn = 12.7:9.1:1.0) (Supplementary Table 2). The lower Li content in o-LISO than that in the initial composition (Li:In:Sn = 17:9:1) confirms Li loss during the heat treatment. In addition, compared with the Li superionic conductivity observed in o-LISO, the RT ionic conductivity in ns-LISO is four orders of magnitude lower, at 3.32 × 10^−8^ S cm^−1^, and the activation energy is much higher, at 552 meV (Supplementary Fig. 8). The ionic conductivity of ns-LISO drops if the calcination time is increased even further, although the DRX phase remains throughout (see Supplementary Note 3).

## Evidence of face-sharing Li configurations in o-LISO

**Fig. 4:**
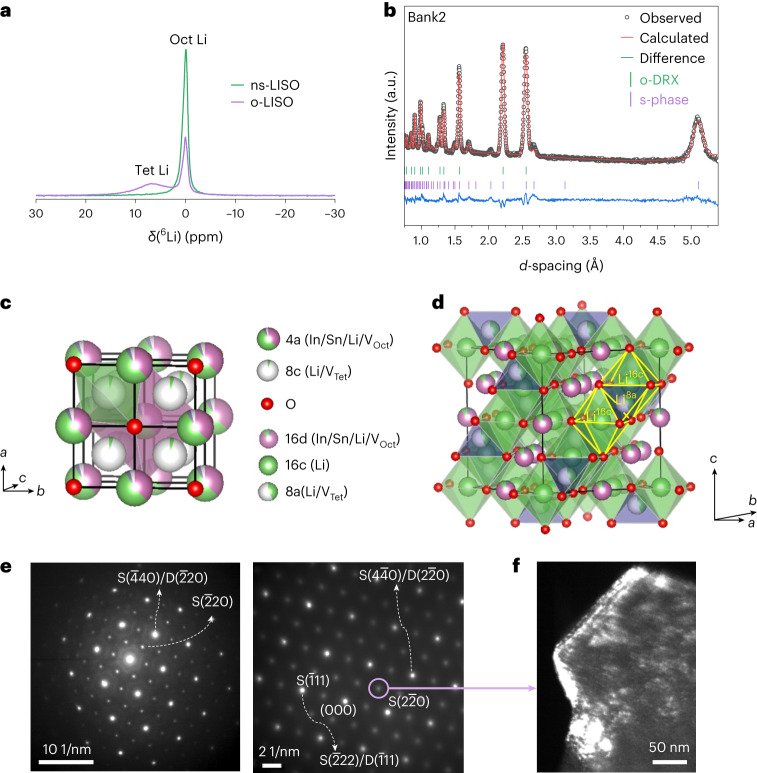
Structural characterization of o-LISO. **a**, ^6^Li MAS ssNMR spectra of o-LISO and ns-LISO at RT and a MAS frequency of 50 kHz. The spectra were scaled according to the amount of powder packed into the rotor and the number of scans. **b**, TOF-NPD pattern (Bank2) of o-LISO and Rietveld refinement. The Bragg positions of o-DRX and the s-phase are marked by green and purple vertical tick marks, respectively. **c**, Structure of o-DRX. V_Tet_, tetrahedral vacancy. **d**, Structure of the s-phase. **e**, TEM electron diffraction patterns collected on o-LISO particles along the zone axes of [111] (left) and [110] (right). Reflections marked with ‘S’ are indexed to the s-phase, and those marked with ‘D’ are indexed to o-DRX. **f**, Dark-field diffraction contrast imaging of an o-LISO particle. Only the electron beams scattered by the s-phase (220) lattice planes (marked by the purple circle in **e**) were selected. Source data

The local Li environment in o-LISO and ns-LISO was probed using ^6^Li ssNMR, with the results shown in Fig. 4a. The ssNMR spectrum of ns-LISO shows a single peak at ~0 ppm, reflecting the Oct Li environment. By contrast, the ssNMR spectrum of o-LISO shows an additional peak at ~6 ppm, which can be attributed to Li in Tet sites. Thus, Li ions in o-LISO occupy both Oct and Tet sites, whereas in ns-LISO only Oct Li is present.

To resolve the detailed structures of o-DRX and the s-phase in o-LISO, Rietveld refinement of time-of-flight neutron powder diffraction (TOF-NPD) data was performed using a two-phase model and a high-throughput grid-search method. As explained in Supplementary Fig. 9 and Note 4, the fit with a two-phase model leads to better results than the single-phase model with selective peak broadening, and is consistent with the dark-field imaging shown later. The refinement results are shown in Fig. 4b and Supplementary Fig. 10, and the detailed methodology of the refinement is described in Supplementary Fig. 11 and Note 5. The resulting structural parameters are given in Supplementary Table 3. Visualization of the structures in Fig. 4c,d shows Li ions occupying the face-sharing Tet and Oct sites in both phases. For o-DRX, Li ions in the Tet site (8c) with an occupancy of 0.058 face-share with the Oct site (4a) occupied by either Li, In or Sn. This Tet Li occupancy is probably the origin of the lattice expansion in o-DRX (Supplementary Fig. 7), as reported when the disordered rocksalt anode Li_3+*x*_V_2_O_5_ is lithiated^21^. Unlike a typical oxide spinel, which has a fully occupied 8a (Tet) site and a completely vacant 16c (Oct) site, the 16c site in the s-phase is fully occupied by Li, and the 8a site is partially occupied by Li with an occupancy of 0.4. Thus, in the s-phase, Li simultaneously occupies the face-sharing 8a and 16c sites, which creates a 3D connected network (Fig. 4d). The identification of Tet–Oct face-sharing Li polyhedra in both phases is consistent with the detection of both Oct and Tet Li environments in o-LISO by ssNMR (Fig. 4a).

The data in Fig. 3 indicate that the formation of the s-phase is induced by over-stoichiometric Li. Within a rocksalt-type framework, over-stoichiometric Li must introduce some degree of the Tet–Oct face-sharing occupancy among cations. We hypothesize that the energy of Tet Li is lower if they face-share only with Li instead of with high-valent In or Sn. This contribution to the energetics will steer the ordering of metal cations away from the cation-disordered state. The cation ordering in the s-phase guarantees that face sharing occurs only among Li occupying 8a and 16c sites, similar to a regular spinel in which the 16c/16d ordering provides the 8a sites with no face-sharing cations. The appearance of the s-phase in o-LISO is thus proposed to be driven by the stabilization of face-sharing Tet Li.

The broad superstructure peaks from the s-phase in the X-ray diffraction patterns (Fig. 3a,b and Supplementary Fig. 1) indicate a small domain size for the cation ordering, which is refined to be 24.5(5) nm (Supplementary Fig. 1). This domain size is much smaller than that of the co-existing o-DRX, of which the X-ray diffraction peaks are sharper and only show instrumental broadening. To understand the distribution of each phase in o-LISO, the microstructure was examined using transmission electron microscopy (TEM). Figure 4e presents the TEM electron diffraction patterns collected on o-LISO particles. Compared with the electron diffraction patterns of ns-LISO with a pure DRX phase (Supplementary Fig. 13), o-LISO shows additional Bragg diffraction spots that indicate unit-cell doubling, consistent with the spinel-like s-phase. Dark-field diffraction contrast imaging was also performed, as shown in Fig. 4f and Supplementary Fig. 14. In this dark-field mode, the s-phase regions appear bright, whereas the o-DRX regions remain dark. The images clearly show that nanosized s-phase domains are dispersed in the o-DRX matrix within the same crystalline particle. The domain size is a few tens of nanometres, consistent with the determination from the synchrotron X-ray diffraction refinement.

## Verified fast Li-ion diffusion with face-sharing configurations

**Fig. 5:**
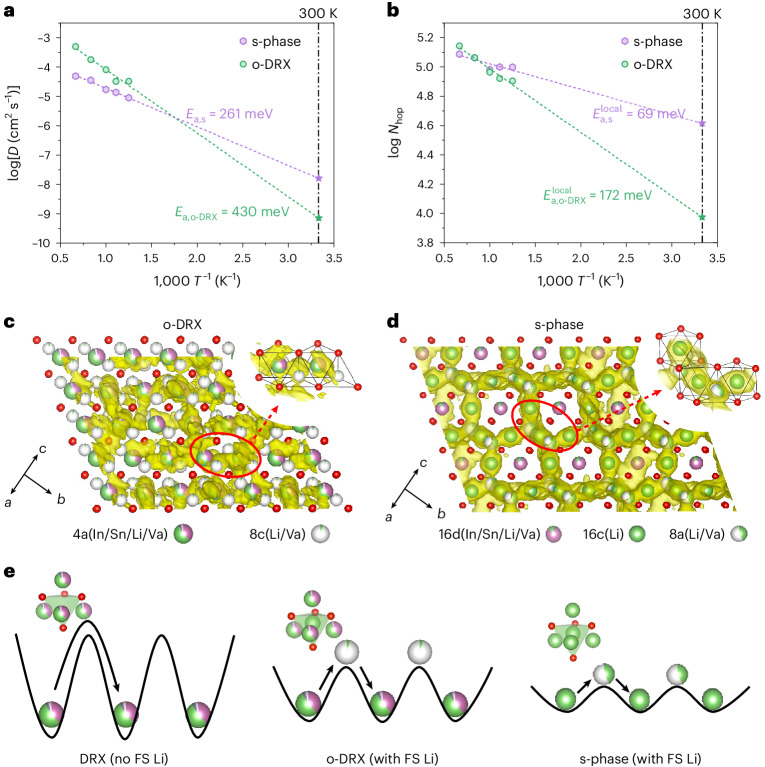
AIMD simulations of o-DRX and the s-phase in o-LISO. **a**,**b**, Arrhenius plots of Li-ion diffusivities from AIMD simulations (**a**) and the number of hops between face-sharing Tet–Oct Li sites during the AIMD trajectory (80 ps) for o-DRX and the s-phase (**b**). **c**,**d**, The calculated Li-ion probability density in o-DRX (**c**) and the s-phase (**d**) from AIMD simulations at 800 K. The atomic structures are overlaid, wherein Li, In, Sn, O and the vacancies (Va) are coloured green, purple, dark purple, red and white, respectively. **e**, Schematic illustrations of the energy landscapes for Li-ion migration in stoichiometric DRX without face-sharing Li (left), o-DRX with face-sharing Li (middle) and the s-phase with face-sharing Li (right). FS, face-sharing. Source data

To more quantitatively connect the face-sharing Li configurations identified in o-LISO to its high ionic conductivity, ab initio molecular dynamics (AIMD) simulations were performed on o-DRX and the s-phase. Details are provided in the Methods. The resulting Li-ion diffusivity (*D*) for each phase is shown in Fig. 5a as function of the temperature. Because diffusivity contains the effect of correlation and percolation, we plot in Fig. 5b the number of hops (*N*_hop_), which more directly indicates the frequency of local hopping between the Tet and Oct sites. The activation energy for Li-ion diffusion in o-DRX is 430 meV, and 261 meV in the s-phase, with extrapolated Li-ion conductivities at 300 K of 1.22 × 10^−4^ S cm^−1^ for o-DRX and 3.17 × 10^−3^ S cm^−1^ for the s-phase. The local hopping barrier (Fig. 5b) is only 69 meV for the s-phase, which is considerably lower than the diffusion activation energy due to the different length and timescale of the ion transport probed. These calculated diffusion and local hopping activation barriers for the s-phase are in excellent agreement with the experimental values obtained for o-LISO (255 meV from EIS, 57–74 meV from ssNMR), suggesting that the fast Li-ion conduction in o-LISO is dominated by ion transport through the s-phase. Therefore, the Li-ion conduction pathways in o-LISO are thought to be primarily through the bulk of the s-phase, with additional pathways through o-DRX to interconnect the s-phase domains, as shown in Supplementary Fig. 16.

The Li-ion density averaged over the AIMD trajectories is visualized in Fig. 5c,d for o-DRX and the s-phase, respectively. Both feature ion hopping through face-sharing Li polyhedra. In a stoichiometric rocksalt structure where all cations occupy Oct positions, the energy for Li-ion hopping through a Tet vacancy is high (Fig. 5e, left) unless no high-valent metal ions occupy the face-sharing sites around the tetrahedron (‘0-TM’), as revealed in studies on DRX cathodes^31,32^. The over-stoichiometry in our materials requires the simultaneous occupancy of face-sharing tetrahedra and octahedra, an effect that will raise the energy of any Li occupying these sites, thereby enhancing their mobility^33^. In addition, Li in a Tet site will prefer to be surrounded by only Li in its face-sharing Oct site to minimize the electrostatic interaction, thereby creating the ‘0-TM’ channels that are typical for spinel-like ordering. The combination of these effects results in a much lower activation energy in o-DRX (430 meV) compared with stoichiometric DRX (552 meV for ns-LISO) (Fig. 5e).

The preference of Tet Li to form into units surrounded by four Oct Li atoms mimics the coordination of the Tet 8a site surrounded by 16c Oct vacancies in a regular spinel. Hence, one can think of the s-phase as a spinel with 8a and 16c occupied by Li, leading to the natural stoichiometry of Li^8a^(Li^16c^)_2_(M^16d^)_2_O_4_ (Li_3_M_2_O_4_), where M denotes a metal. The fact that our Li over-stoichiometry does not reach this level indicates that the spinel-like ordering is incomplete. Compared with o-DRX, where the face-sharing Li polyhedrons may be isolated, the s-phase has all of the Tet Li (8a) in 0-TM channels and all of the face-sharing Li 3D connected. The difference in local hopping energy (Fig. 5b) between o-DRX and the s-phase confirms the importance of these 0-TM configurations for fast hopping through the Tet site. Therefore, we observe more 3D percolating ion-diffusion pathways in the s-phase than in o-DRX (Fig. 5c,d), and the ion-migration barrier in the s-phase is further lowered (Fig. 5e, right). The high Li-ion conductivity in both o-DRX and the s-phase indicates that the face-sharing Li configurations can generally improve the Li-ion mobility in fcc-type oxides.

## Compositional design guidelines for over-stoichiometric rocksalt-type compounds

**Fig. 6:**
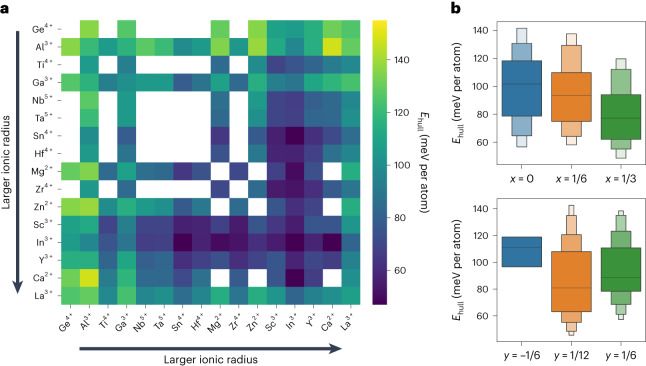
High-throughput computational phase-stability analysis. **a**, Metal-compatibility heatmap extracted from high-throughput DFT calculations. The average *E*_hull_ values among all the considered structural configurations (γ-LiFeO_2_-like ordering, spinel-like ordering, layered-like ordering and the electrostatic ground state of the cation arrangement) for the selected binary metal chemical spaces were averaged over all computed compositions with the Li-excess level *x* = 0, 1/6, 1/3 and the Li over-stoichiometry level *y* = 1/12, 1/6. The metal cation species are arranged according to their ionic radii in the axes. Locations without a pixel indicate that the corresponding M1–M2 pair cannot satisfy the considered *x* and *y* due to valence constraints. Darker blue indicates lower *E*_hull_ values and greater stability, and brighter green indicates poorer stability. **b**, Probability density distribution of *E*_hull_ for all computed compounds at different Li-excess levels (*x*, top) and Li over-stoichiometric levels (*y*, bottom). A negative *y* indicates Li vacancies at Oct sites. In the boxen plots, the centre line represents the median and each successive level outwards contains half of the remaining data. Source data

The success in constructing face-sharing Li configurations and the realization of high Li-ion conductivity in o-LISO motivated us to explore a broader M1–M2 compositional space beyond the In–Sn system to create ORX-based SICs. To investigate compositions that may stabilize the presence of Li over-stoichiometry, we performed the high-throughput screening of ORX compositions via density functional theory (DFT) calculations. We selected 16 redox-inactive metal species for making rocksalt-type Li_1+*x*+2*y*_M1_*z*_M2_1−*x*−*z*_O_2_ compositions, where *y* is the Li over-stoichiometry level that makes the overall cation/anion ratio larger than 1, *x* is the Li-excess level that leads to Li substitution of other metal cations in Oct sites while maintaining the cation/anion ratio and *z* can be calculated based on charge neutrality. The energy stability of each composition is evaluated by analysing its competing phases and calculating the corresponding average energy above the hull (*E*_hull_) from all of the considered structural configurations. More computational details can be found in the Methods. Figure 6a presents a metal-compatibility heatmap, where each pixel represents the average *E*_hull_ of the computed compositions for the selected metal pairs. A darker blue colour represents a lower average *E*_hull_, indicating that the corresponding M1–M2 couple can better accommodate the face-sharing configurations created by Li over-stoichiometry. From Fig. 6a we find that using larger metal cations (for example, In^3+^) tends to result in greater stability, with an average *E*_hull_ below 80 meV per atom. In a rocksalt structure, the ionic radius of the octahedrally coordinated metal cation usually determines the volume of its face-sharing Tet vacancy^34^. Larger octahedral cations create a more spacious Tet site for Li to reside in and thus stabilize Li over-stoichiometry. We note that La^3+^ is an exception here as it is too large to stabilize a six-fold coordination with oxygen^35^.

To further deconvolute the effect of Li content on the stability of the ORX compounds, we investigated the probability distribution of *E*_hull_ for all of the computed chemistries at the considered levels of Li excess (*x*) and Li over-stoichiometry (or Li vacancy) (*y*), as plotted in Fig. 6b. From the lower panel, we find the probability density distribution centres to be at lower median *E*_hull_ values for compositions with Li over-stoichiometry (*y* = 1/12, 1/6) compared with those with Li vacancies at Oct sites (*y* = −1/6). This indicates that a low-to-intermediate Li over-stoichiometry level is easier to be incorporated into the rocksalt lattice than Li vacancies. As shown in the upper panel, a higher Li-excess level monotonically shifts the *E*_hull_ distribution downwards in the given range of *x*, indicating that the Li excess helps to stabilize the over-stoichiometric Li present in the system. This role of Li excess may be attributed to its electrostatic modifications on the local face-sharing environment. Specifically, a greater Li excess lowers the average valence of the octahedral cations and introduces a greater occurrence of Li occupying face-sharing Oct sites around Tet Li or even 0-TM channels, thus reducing the electrostatic repulsion felt by Tet Li^31^. Therefore, large redox-inactive metal cations (for example, In^3+^) and a high Li-excess level are key to stabilizing ORX compounds with face-sharing Li configurations.

## Outlook for over-stoichiometric rocksalt-type SICs

The local cation configuration plays a vital role in achieving a high Li-ion conductivity, especially for oxides that have weaker anion polarizability than the heavier anion chemistries such as S, Cl and Br. Despite being the most common anion packing for oxides, the fcc arrangement of oxygen ions has traditionally been considered to be unfavourable for fast ion conduction. Our strategy has been to introduce face-sharing cations by introducing Li over-stoichiometry, giving Li a higher mobility. The ordering of Li_Tet_(Li_Oct_)_4_ face-sharing clusters into a spinel-like arrangement provides good percolation and 3D connectivity of the activated Li ions. The resulting ORX compound, o-LISO shown in this work, exhibits a Li-ion conductivity of 3.38 × 10^−4^ S cm^−1^ at ambient temperature with a low activation energy of 255 meV, which is dramatically superior compared with that of fcc-type oxides without face-sharing Li. Considering the remarkable robustness of the rocksalt structure to chemical variation, the discovery of ORX-based SICs substantially expands the scope of potential solid-state electrolytes for ASSBs. As an extension of the In–Sn compound introduced in this work, we also experimentally prepared ORX compounds in the In–Mg, In–Zn and In–Ti chemical spaces. All of these ORX compounds show similar s-phase formation with Li over-stoichiometry and exhibit notably improved Li-ion conductivities that reach between 10^−5^ and 10^−4^ S cm^−1^ at RT (Supplementary Fig. 17 and Note 6). Furthermore, we have pointed out that the use of large redox-inactive metal cations and high Li-excess levels are key to stabilizing the face-sharing Tet Li in future design of ORX-type SICs.

The observation of spinel-like ordering in an over-stoichiometric rocksalt is at first surprising given that spinel is normally found for cation/anion ratios close to 0.75. Our finding of the new s-phase illustrates the isomorphism between the s-phase and regular spinel once one considers the equivalence between 16c vacancies in regular spinel and Li-occupied 16c in the s-phase. In a regular spinel, the occupancy of the 8a Tet site forces 16c vacancies, creating a ‘spinel unit’ that consists of one 8a and four 16c sites. In the s-phase, this unit is created by the preference of a Tet Li to have four Li ions in its face-sharing Oct. The spinel is, ultimately, the regular ordering of these units. This unique s-phase structure leads to a 3D fully percolated Li-ion diffusion pathway composed of 8a–16c–8a face-sharing polyhedra, resulting in a high Li-ion conductivity of 3.17 × 10^−3^ S cm^−1^ at 300 K in AIMD simulations. We observe that the s-phase forms as nanosized domains in the o-DRX matrix. The co-existence of the s-phase and o-DRX is thus merely the result of different cation orderings within the same continuous anion sublattice.

Through cyclic voltammetry tests and interfacial chemical reaction energy calculations (Supplementary Fig. 18, Table 4 and Note 7), we further evaluated that o-LISO shows great oxidative stability (up to 4 V) and excellent chemical stability with known cathodes (for example, LiCoO_2_, Li(NiMnCo)_1/3_O_2_), although its poor stability with Li metal due to the easy reduction of In^3+^ needs to be further addressed by replacing In^3+^ with other more reductively stable cations or by appropriate boundary layers.

In summary, we demonstrate that incorporating excess Li to form cation over-stoichiometry in rocksalt-type lattices is an effective approach to create face-sharing Li configurations, yielding Li superionic conductivity in an fcc-type oxide. Such Li over-stoichiometry is achievable through straightforward solid-state synthesis and induces local spinel-like metal ordering to reduce the electrostatic repulsion between face-sharing sites. As a result, the s-phase with 3D fully connected face-sharing Li polyhedra forms as nanosized domains in the o-DRX matrix. Furthermore, we show that large metal cations and a substantial Li excess are beneficial for accommodating the over-stoichiometric Li and stabilizing the face-sharing Li configurations. Our discovery provides guidelines and unlocks the potential for designing Li SICs within the vast chemical space of fcc-type oxides.

## Methods

### Synthesis

All of the Li–In–Sn–O (LISO) compounds were synthesized using a traditional solid-state method. Li_2_CO_3_ (Alfa Aesar, ACS, 99% minimum), In_2_O_3_ (Sigma-Aldrich, 99.998%) and SnO_2_ (Sigma-Aldrich, 99.9%) were used as the precursors. All of the precursors were stoichiometrically mixed in ethanol (except that 10% excess Li_2_CO_3_ was added to compensate for the Li loss during synthesis) in a 50 ml stainless steel jar with five 10-mm-diameter stainless steel balls using a Retsch PM 200 planetary ball mill at 250 revolutions per min for 12 h. The precursors were then dried overnight in an oven at 70 °C before being pelletized. The precursor pellets were first calcinated in air at 1,050 °C and then air-quenched to RT. After the calcination, the pellets were manually ground into powders and shaker-milled for 30 min in air using a SPEX 8000M mixer/mill to decrease the particle size. The resulting powders were pelletized and sintered in air again at 1,050 °C to densify the pellets. The sintered pellets were air-quenched to RT and transferred to a glovebox for further study. The calcination and sintering times, respectively, were 4 and 6 h for o-LISO and 6 and 10 h for ns-LISO. The calcination and sintering times must be well controlled to ensure the appropriate amount of Li loss, and they can be dependent on the furnace because it affects the rate of Li loss. The key is to always add some excess Li precursor at the beginning and to use the heat treatment time to control the Li loss to the desired amount. For example, for o-LISO, we wanted to achieve the Li content at which DRX and the s-phase appeared without the Li_3_InO_3_-like phase.

### Conductivity measurements

The Li-ion conductivities were evaluated using EIS with indium (In) metal as the ion-blocking electrodes at temperatures ranging from 0 to 120 °C. The sintered pellets (at ~90–95% relative density; see Supplementary Fig. 15 for scanning electron microscopy (SEM) cross-sectional images) were first polished with sandpaper to remove the surface layer that had severe Li loss and then sandwiched between two In films. The pellets with In films were pressed using cold isostatic pressing (using a YLJ-CIP-20B press (MTI)), to ensure good contacts between the In films and the pellet, and then transferred to Bio-Logic leak-tight sample holders (CESH) for the EIS measurements. The EIS measurements were performed using an EC-Lab Electrochemistry VMP300 instrument (Bio-Logic) in the frequency range of 7 MHz to 100 mHz with a 10 mV voltage amplitude. A Bio-Logic intermediate temperature system was used to control the temperature of the sample holder. EIS data fitting was performed using the ZView software package^36^. The electronic conductivity was evaluated using a direct-current polarization test with In metal as the electrodes.

### Electrochemistry

For cyclic voltammetry tests, a Li/liquid electrolyte/o-LISO-C cell was assembled. To make the o-LISO-C films, as-synthesized o-LISO, carbon nanofibres and polyvinylidene fluoride were mixed and dispensed in *N*-methyl-2-pyrrolidone with a weight ratio of 7:2:1, respectively, to make the electrode slurry. The electrodes were prepared by casting the electrode slurry onto aluminium foils before drying in a 70 °C vacuum oven overnight. Discs (6 mm in diameter) with the loading of o-LISO about 2 mg were punched from the electrode films and assembled into a liquid coin cell using Li-metal foil as the counter electrode and commercial 1 M LiPF_6_ in ethylene carbonate and dimethyl carbonate solution (1:1 volume ratio) as the electrolyte. The cyclic voltammetry measurements were performed at a scan rate of 0.1 mV s^−1^ using the VMP300 instrument.

### Characterization

The laboratory X-ray diffraction patterns of the as-synthesized compounds were obtained using a Rigaku MiniFlex 600 diffractometer equipped with a Cu source. The synchrotron X-ray diffraction pattern of o-LISO was obtained at Beamline 17-BM at Argonne National Laboratory. The TOF-NPD experiments on o-LISO were performed at the Spallation Neutron Source at Oak Ridge National Laboratory using the Nanoscale Ordered Materials Diffractometer^37^. Rietveld refinement^38^ was performed using the GSAS-II software package^39^ and a high-throughput grid-search method (the details are described in Supplementary Fig. 11 and Note 5). Elemental analyses were performed by Galbraith Laboratories using inductively coupled plasma optical emission spectroscopy for the Li, In and Sn species. SEM images were collected using a Zeiss Gemini Ultra-55 analytical field-emission scanning electron microscope at the Molecular Foundry at Lawrence Berkeley National Laboratory. Thermogravimetric analysis was performed using a Q600 SDT instrument under O_2_ flow.

### Solid-state NMR spectroscopy

Both RT and variable-temperature ssNMR data on o-LISO and ns-LISO powder samples were acquired using a Bruker Avance IIIHD 700 MHz (16.4 T) NMR spectrometer with a Larmor frequency of 103.03 MHz for ^6^Li. The ^6^Li spin echo RT spectra for o-LISO and ns-LISO were acquired using a 90° radiofrequency (RF) pulse of 3.75 μs and a 180° RF pulse of 7.5 μs at 50 W using 50 kHz MAS and a 1.3 mm double-resonance HX probe. A recycle delay of 5 s was used. Variable-temperature ^6^Li ssNMR data were acquired for o-LISO at 12.5 kHz MAS using a 4 mm triple-resonance HXY probe. The ^6^Li spin echo spectra were acquired using a 90° RF pulse of 6.2 μs and a 180° RF pulse of 12.4 μs at 300 W. Spin–lattice (*T*_1_) relaxation time constants were measured using a saturation recovery experiment and spin–spin (*T*_2_) relaxation time constants were measured using a variable-delay Hahn echo experiment. The temperature was calibrated using the ^79^Br chemical shift of KBr. The spectra were referenced against ^6^Li-enriched Li_2_CO_3_ (*δ*(^6^Li) = 0 ppm). Spectral deconvolution was performed using the DMFIT software package^40^.

### TEM characterization

TEM samples of o-LISO and ns-LISO were prepared by dispersing the particles onto TEM lacey carbon grids inside an argon-filled glovebox. Diffraction contrast imaging and energy-dispersive X-ray spectroscopy (EDS) were conducted using a monochromated aberration-corrected Titan 80-300 scanning transmission electron microscope operated at 300 kV. To obtain the dark-field diffraction contrast images, the objective aperture was inserted in the back focal plane to select the electrons scattered only from ($2\bar{2}0$)_s_ planes. EDS data were collected using a Super-X detector inside a Themis Z scanning transmission electron microscope operated at 300 kV. The EDS data analysis was performed using Velox software (ThermoFisher Scientific), where the overlapped peaks were deconvoluted using the stored standard reference spectra and using a Filtered Least Squares method to fit the peaks and remove the background.

### DFT calculations

First-principles total energy calculations were performed using the Vienna ab initio simulation package with a plane-wave basis set^41^. Projector augmented-wave potentials^42^ with a kinetic energy cutoff of 520 eV and the exchange-correlation form in the Perdew–Burke–Ernzerhof generalized gradient approximation^42^ were used in all of the structural optimizations and total energy calculations. For all of the calculations, a reciprocal space discretization of 25 k-points per Å^−1^ was applied, and the convergence criteria were set to 10^−6^ eV for electronic iterations and 0.02 eV Å^−1^ for ionic iterations.

For the high-throughput phase-diagram calculations, ORX compositions with Li-excess levels of *x* = 1/3, 1/6 or 0 and Li over-stoichiometric levels of *y* = 1/6 or 1/12, and compositions with a Li-excess level of 1/3 and 1/6 intrinsic Li vacancy (*y* = −1/6) were considered. Sixteen metal cations that are redox-inactive and commonly used in solid-state electrolytes were selected, for example, Mg^2+^, Zn^2+^, Ca^2+^, Al^3+^, Ga^3+^, In^3+^, Sc^3+^, Y^3+^, La^3+^, Ge^4+^, Ti^4+^, Sn^4+^, Zr^4+^, Hf^4+^, Ta^5+^ and Nb^5+^. For each chemical composition, four different orderings, including γ-LiFeO_2_ ordering, spinel-like ordering, layered-like ordering and the electrostatic ground state, were considered. For each type of ordering, we enumerated ten configurations that have a near-ground-state electrostatic Ewald energy for DFT calculation. The thermodynamic stability of all of the ORX compositions was evaluated by constructing the convex hull of the DFT total energy for all phases in the relevant chemical space available in an internal database that contains phases from the Inorganic Crystal Structure Database and some compounds generated using data-mined substitution rules^43^. The convex hull ensures that each ground state has an energy lower than any linear combination of phases that leads to the same composition as the ground state. The phase stability for phases not on the hull was quantified via their *E*_hull_, which indicates the compound’s driving force for decomposition into other ground states. The *E*_hull_ serves as a reasonable indicator of synthetic accessibility, as experimentally accessible materials must generally have a low *E*_hull_ (refs. ^44,45^).

The interfacial chemical reaction energy was calculated based on the methodology of Richards and co-workers^8^. For two crystalline reactants, A and B, there exists a number of possible reactions that consume arbitrary compositions of either phase (*c*_A_ and *c*_B_) to form lower-energy phase equilibrium products, $x{c}_{{\rm{A}}}+\left(1-x\right){c}_{{\mathrm{B}}}\to {c}_{{\rm{equil}}}$. The reaction energy Δ*E*_rxn_ was obtained by evaluating the mixing ratio *x* that yielded the largest reaction driving force, as shown in the following equation:

$$\Delta {E}_{{\rm{rxn}}}=\mathop{\min }\limits_{x\in \left[0,1\right]}\left\{{E}_{{\rm{pd}}}\left[x{c}_{{\rm{A}}}+\left(1-x\right){c}_{{\rm{B}}}\right]-{xE}\left[{c}_{{\rm{A}}}\right]-\left(1-x\right)E\left[{c}_{{\rm{B}}}\right]\right\}.$$

Here, the compositions *c*_A_ and *c*_B_ are normalized by their number of atoms, and the function *E*_pd_[*c*] describes the energy of the ground-state structure or phase equilibrium at composition *c* determined from phase diagrams and convex hull based on computed entries in the Materials Project database^46^.

### AIMD simulations

AIMD for both o-DRX and the s-phase were performed to investigate the ionic conductivity. For both o-DRX and the s-phase, we created Oct site ordering reflecting a DRX and spinel-like structure (a low-temperature LiCoO_2_ structure). The occupancies of both the Tet and Oct sites were then modified to reach our target composition. After the partially disordered structures were established, we performed structural enumeration and choose ten configurations with low Ewald electrostatic energy values for DFT calculations. The resulting lowest energy structure for each phase was used for the AIMD simulations. All of the AIMD calculations were performed in an *NVT* ensemble (where *N* is the number of particles in the system, *V* is the system volume and *T* is the absolute temperature) with a time step of 1 fs and using a Nosé–Hoover thermostat^47^ for a period of 100 ps. The diffusion barrier was calculated via Arrhenius fitting of the diffusivity at different temperatures. The activation barrier of local hopping was calculated from the Arrhenius fitting of the local hopping amounts at different temperatures. The local hopping amounts were calculated by counting the number of jumps from the Oct site to the Tet site or vice versa.

## Online content

Any methods, additional references, Nature Portfolio reporting summaries, source data, extended data, supplementary information, acknowledgements, peer review information; details of author contributions and competing interests; and statements of data and code availability are available at 10.1038/s41563-024-01800-8.

### Supplementary information

Supplementary Information Supplementary Figs. 1–18, Notes 1–7, Tables 1–4 and refs. 1–19.

### Source data

Source Data Fig. 2 Variable-temperature EIS and ssNMR data.

Source Data Fig. 3 X-ray diffraction data.

Source Data Fig. 4 Solid-state NMR spectra and neutron powder diffraction data.

Source Data Fig. 5 AIMD simulation data.

Source Data Fig. 6 Compositional high-throughput DFT calculation data.

## Data Availability

All data that support the findings in this study are available within this article and its Supplementary Information. Any additional relevant data are available from the corresponding authors upon request. Source data are provided with this paper.
