# Publisher Correction: Unlocking Li superionic conductivity in face-centred cubic oxides via face-sharing configurations

**DOI:** 10.1038/s41563-024-01878-0

**Published:** 2024-03-27

**Authors:** Yu Chen, Zhengyan Lun, Xinye Zhao, Krishna Prasad Koirala, Linze Li, Yingzhi Sun, Christopher A. O’Keefe, Xiaochen Yang, Zijian Cai, Chongmin Wang, Huiwen Ji, Clare P. Grey, Bin Ouyang, Gerbrand Ceder

**Affiliations:** 1 Department of Materials Science and Engineering, University of California, Berkeley, CA USA; 2 Materials Sciences Division, Lawrence Berkeley National Laboratory, Berkeley, CA USA; 3 Yusuf Hamied Department of Chemistry, University of Cambridge, Cambridge, UK; 4 School of Chemical Sciences, University of Chinese Academy of Sciences, Beijing, China; 5 Physical and Computational Sciences Directorate, Pacific Northwest National Laboratory, Richland, WA USA; 6 Environmental Molecular Sciences Laboratory, Pacific Northwest National Laboratory, Richland, WA USA; 7 Department of Materials Science and Engineering, University of Utah, Salt Lake City, UT USA; 8 Department of Chemistry and Biochemistry, Florida State University, Tallahassee, FL USA

**Keywords:** Batteries, Ceramics, Solid-state chemistry

Correction to: *Nature Materials* 10.1038/s41563-024-01800-8, published online 2 February 2024.

In the version of the article initially published, the *x* axes in Figs. 2b,d and 5a,b originally read “*T*^−1^ (K^−1^)” and have now been amended to “1000 *T*^−1^ (K^−1^)” in the HTML and PDF versions of the article.

