# Supplementary material for: Unlocking Li superionic conductivity in face-centred cubic oxides via face-sharing configurations
Source: Nat Mater. 2024 Feb 2;23(4):535–42. doi: 10.1038/s41563-024-01800-8 (PMC10990923; doi:10.1038/s41563-024-01800-8)
Supplement: Supplementary file 1 — Supplementary Figs. 1–18, Notes 1–7, Tables 1–4 and refs. 1–19. [file 41563_2024_1800_MOESM1_ESM.pdf]

nature materials

Article

https://doi.org/10.1038/s41563-024-01800-8

Unlocking Li superionic conductivity in face-centred cubic oxides via face-sharing configurations

In the format provided by the authors and unedited

Supplementary information

Table of Contents

Supplementary Figs. 1-18

Supplementary Notes 1-7

Supplementary Tables 1-4

Supplementary References

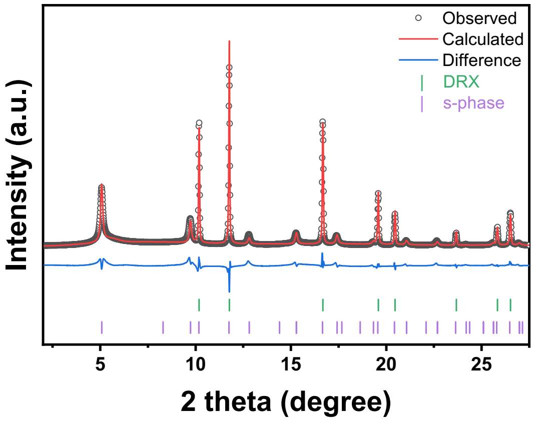

Figure S1. Rietveld refinement of synchrotron XRD of o-LISO using a two-phase model. R_{wp} = 13.62%

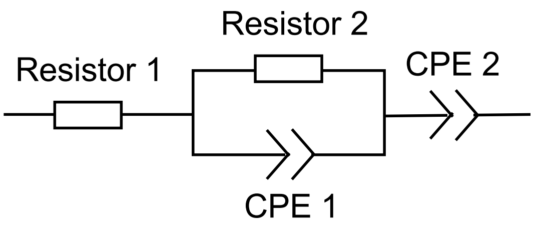

	Value
Resistor 1	20.05 ohm
Resistor 2	852.3 ohm
CPE 1-Q	6.12×10^{-10} F
CPE1-α	0.896
CPE 2-Q	5.48×10^{-6} F
CPE 2-α	0.604

Figure S2. The equivalent circuit model used for the fitting of EIS spectrum of o-LISO and the resulting fitting parameters.

Supplementary Note 1: Details of EIS fitting

A simple equivalent circuit model shown in the Figure S2 was used for the fitting of EIS spectrum, which has been frequently used for pure ionic conductors[1]. The constant phase element (CPE) is typically used to model the behavior of a double layer that is an imperfect capacitor. The parallel resistor and CPE (Resistor 2//CPE 1) elements were used to fit the semicircle in the Nyquist plot[2]. Since only a single semicircle was observed in the Nyquist plot of o-LISO, the grain (bulk) and grain boundary contributions cannot be deconvoluted. The capacitance of CPE 1, 6.12×10^{-10} F, implies the process stems from both grain and grain boundary contributions[3]. Thus, the resistance of Resistor 2 represents a sum of the bulk and grain boundary resistances, which was used to calculate the total ionic conductivity. The CPE 2 modeled the linear spike at low-frequency region for cells measured in an ion-blocking configuration, which is attributed to the accumulation of Li-ions at the interface.

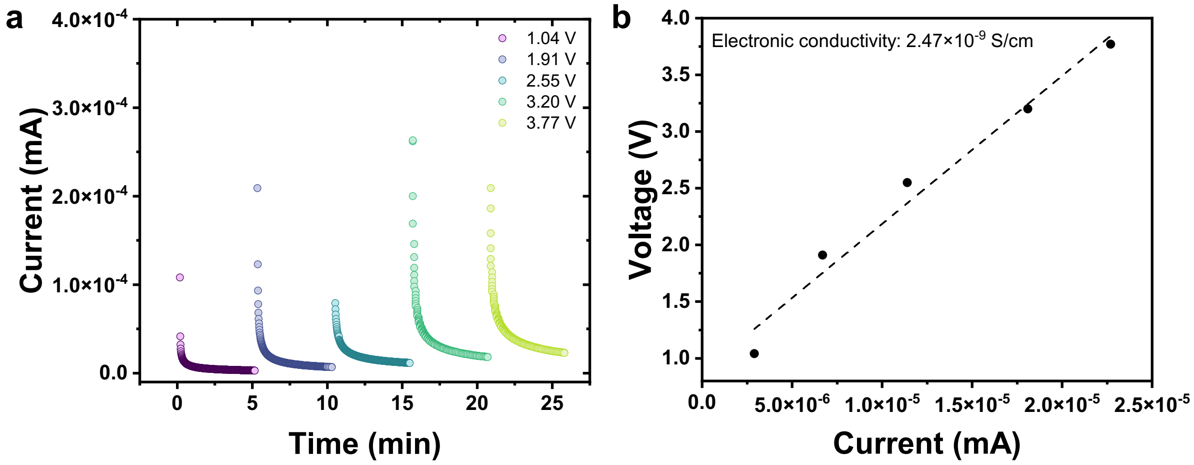

Figure S3. **(a)** The d.c. polarization curves of o-LISO using ion-blocking electrodes at different voltages. The steady current value at the end of the curve was used for the calculation of electronic resistance. **(b)** Voltage versus current curve from d.c. tests, the slope from linear fitting was used to calculate electronic conductivity based on ohm's law.

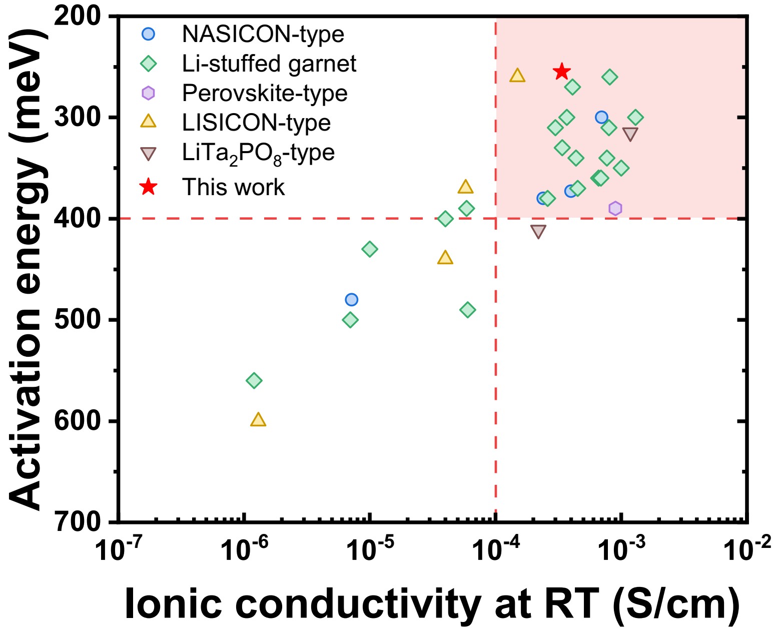

Figure S4. Ion conducting properties of o-LISO (this work) versus known oxide-based solid-state Li conductors (experimental reports)[4-16]. The red-shaded region refers to superionic conductors, which typically have room-temperature ionic conductivities higher than 10^{-4} S cm^{-1} at room temperature and activation energies for ion conduction lower than 400 meV.

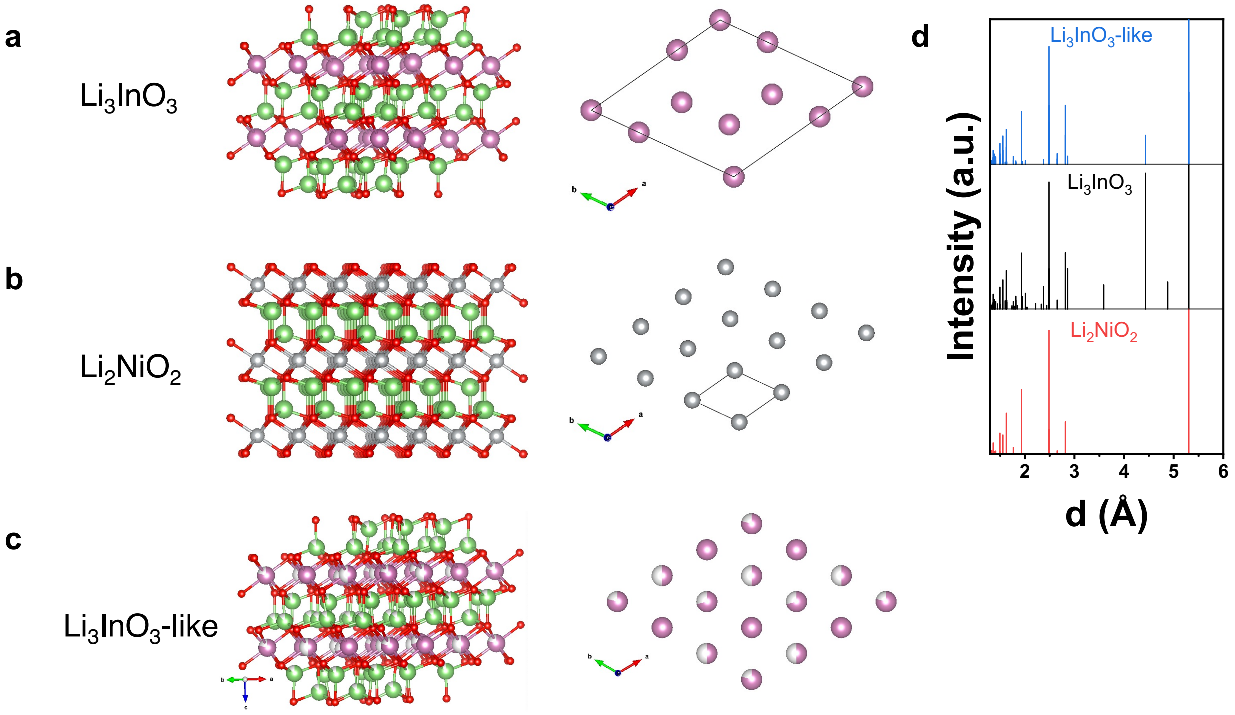

Figure S5. Crystal structures (left) and views of the non-Li metal layer down the c axis (right) of **(a)** Li_3InO_3, **(b)** 1T-Li_2NiO_2, **(c)** the Li_3InO_3-like phase formed in LISO with excessively over-stoichiometric Li. The Li, In, O, Ni atoms are represented by green, purple, red, and grey colors. **(d)** The calculated XRD patterns for the three phases. Note that the lattice parameters of the three phases are matched manually for the easier comparison of the XRD patterns.

Supplementary Note 2: Elaboration of the structure of Li_3InO_3-like phase

The Li_3InO_3-like phase observed during the synthesis has a similar structural framework as Li_3InO_3 and 1T-Li_2NiO_2 phases. The structures of Li_3InO_3 and 1T-Li_2NiO_2 are shown in Figs S5a-b. They have both layered structures with In/Ni layer and Li dumbbell layer. The differences are that in 1T-Li_2NiO_2, all the Oct sites in the Ni layer are occupied, while in Li_3InO_3, there are 1/3 Oct vacancies in the In layer. This leads to the symmetry breaking in Li_3InO_3 compared to that of 1T-Li_2NiO_2, resulting in more reflection peaks in the XRD pattern for Li_3InO_3 (Figs. S5d). If we

compare the experimentally obtained XRD pattern of Li_3InO_3-like phase (Fig. 3) with those of Li_3InO_3 and 1T-Li_2NiO_2, we can see that the symmetry of Li_3InO_3-like phase is in between. Based on the observed XRD pattern for Li_3InO_3-like phase, we roughly refined the structure and found that Oct vacancies in the In layer is less than that in Li_3InO_3 phase, as shown in Fig. S5c. In other words, in Li_3InO_3-like phase, more than 2/3 Oct sites in the In layer are occupied by In/Sn, which also leads to the vacancies in the Tet sites in the Li dumbbell layer to keep the charge balance. However, the fine structure of Li_3InO_3-like phase cannot be refined because it only forms as a small impurity phase in the LISO sample with excessively over-stoichiometric Li. Since it doesn't have the desired face-sharing Li configuration, we think this phase is less of interest and didn't do a further study.

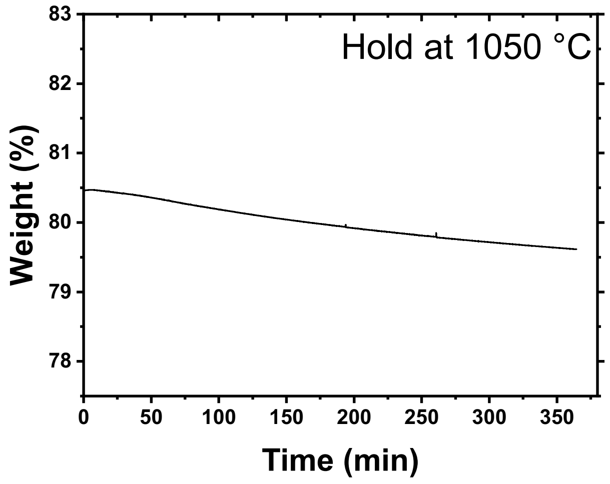

Figure S6. Thermogravimetric analysis (TGA) of the LISO17 sample upon sintering at 1050 °C. The weight percentage is normalized by the precursor weight before calcination.

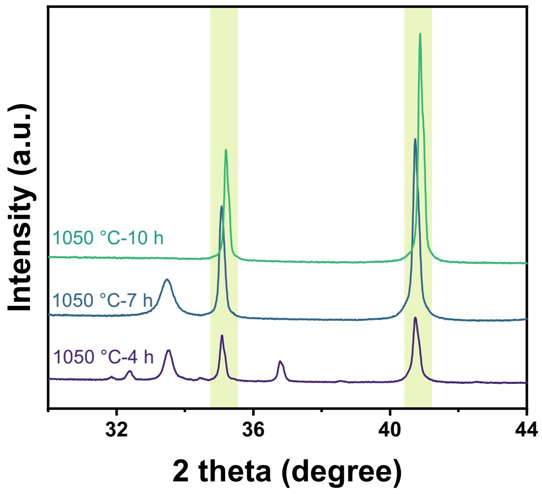

Figure S7. The enlarged XRD patterns of LISO17 samples with the calcination time of 4 h (excessively over-stoichiometric), 7 h (over-stoichiometric), and 10 h (near-stoichiometric). The peaks in the shaded regions are from DRX phase.

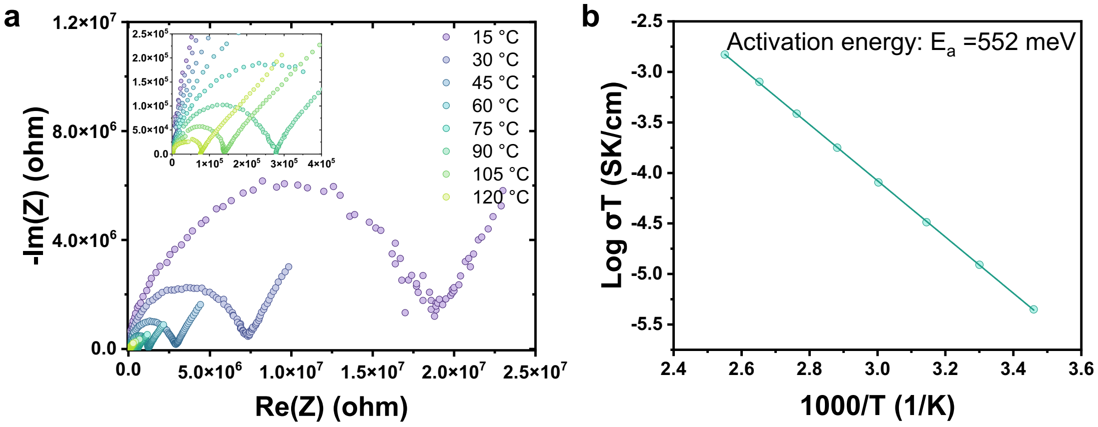

Figure S8. (a) Nyquist plots of ns-LISO at variable temperatures from 15 °C to 120 °C. **(b)** Arrhenius plots of ionic conductivity values obtained from variable temperature EIS measurements for ns-LISO.

Supplementary Note 3: Li-ion conductivity of ns-LISO

The ns-LISO reported in the main text was synthesized by calcinate at 1050 °C for 6 h and then sintered at 1050 °C for 10 h. If we apply a longer calcination or sintering time, e.g. 12h, that DRX phase is maintained, the ionic conductivity can be even lower and lower than the detection limit by EIS. This is likely due to the further Li-loss with longer calcination or sintering time. This phenomenon also suggest that the Li content affect the ionic conductivity a lot.

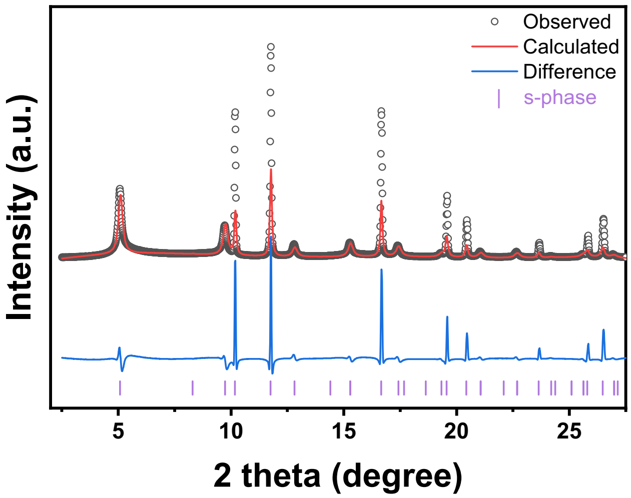

Figure S9. Rietveld refinement of synchrotron XRD of o-LISO using a single-phase model with selective peak broadening, which shows a poor fitting result.

Supplementary Note 4: Rationale of using two-phase model in Rietveld refinement

The XRD pattern of o-LISO exhibits broad peaks characteristic of the s-phase and sharp peaks that are common to the o-DRX and s-phase. As the diffraction peaks of the o-DRX phase fully overlap with those of s-phase, it is not trivial to determine if the o-DRX and s-phase co-exist with different domain size, or whether a single s-phase with small domain size leads to selective peak broadening. To differentiate these two scenarios, we tried refining the synchrotron XRD data using a single spinel-like phase with selective peak broadening. This refinement method has been reported in a previous study[17]. The result is shown in Fig. S9. Although the selective peak broadening can fit the broad peaks characteristic of s-phase, a large intensity disparity between calculated and observed curves is observed for those sharp peaks overlapping with o-DRX phase. This suggests that a single s-phase is insufficient to fit the diffraction pattern, confirming the presence of a DRX phase. Therefore, in the Rietveld refinement of synchrotron XRD and TOF-NPD data, we chose a two-phase model.

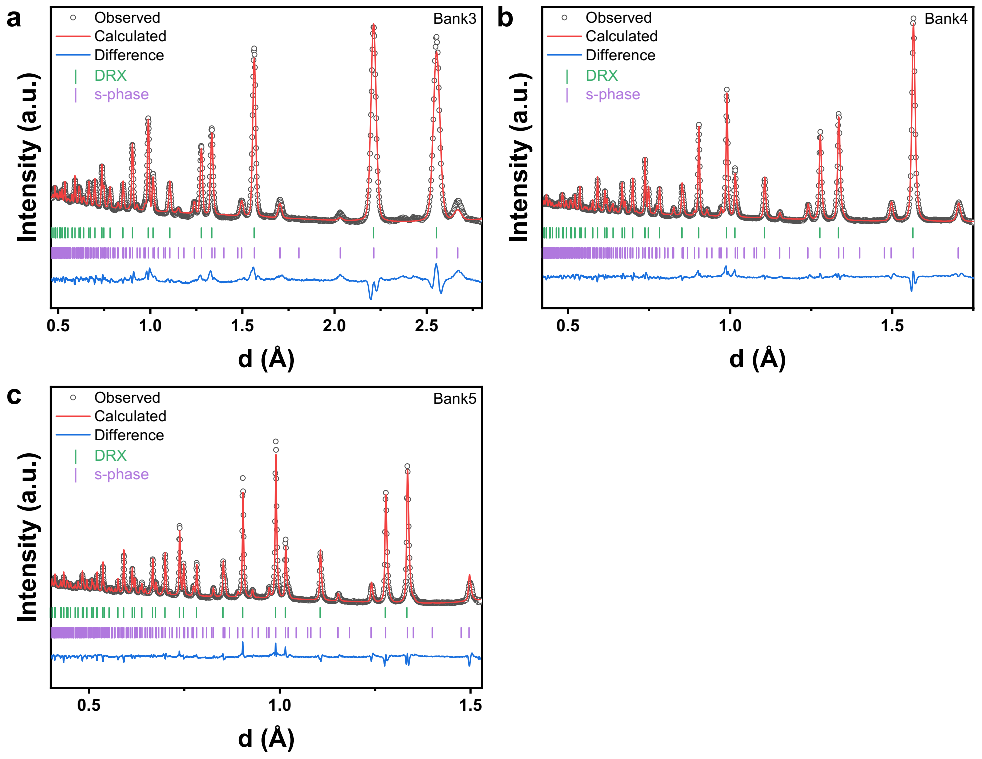

Figure S10. Rietveld refinement of time-of-flight neutron powder diffraction patterns of **(a)** Bank 3, **(b)** Bank 4, **(c)** Bank 5 for o-LISO. Calculated positions of Bragg reflections of o-DRX and s-phase are shown by green and purple vertical tick marks, respectively.

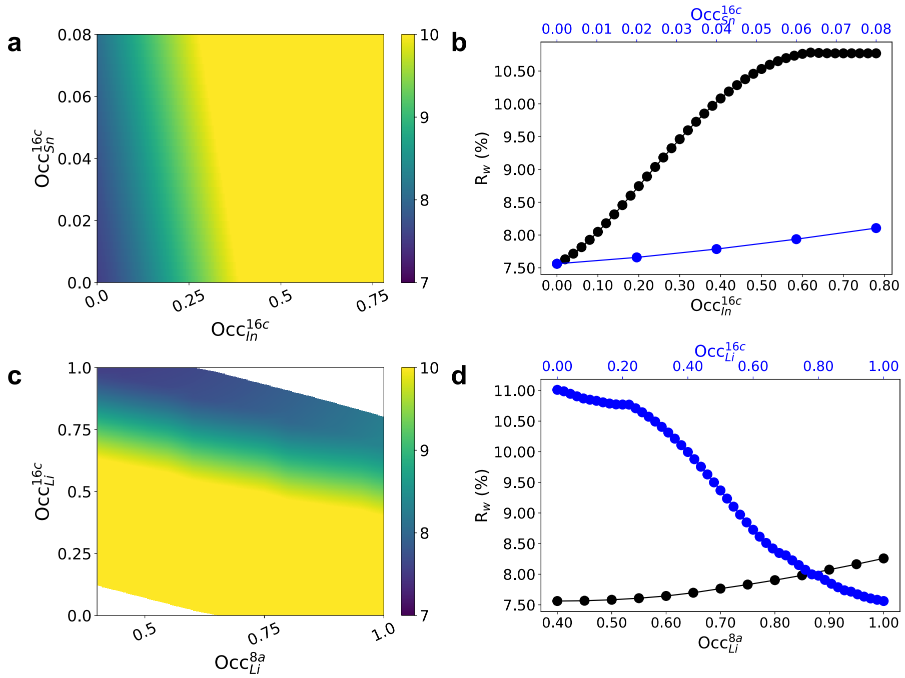

Figure S11. (a) R-factor mapping with variable Occ_{In}^{16c} and Occ_{Sn}^{16c} of s-phase from the 4D high throughput grid search. The R_{wp} value at given Occ_{In}^{16c} and Occ_{Sn}^{16c} is calculated by taking the minimum R_{wp} from all possible 8a and 16c Li occupancies in s-phase. **(b)** The minimum R_{wp} value as a function of $Occ_{In}^{16c}/Occ_{Sn}^{16c}$. **(c)** R-factor mapping with variable Occ_{Li}^{16c} and Occ_{Li}^{8a} of s-phase from the 4D high throughput grid search. The R_{wp} value at given Occ_{Li}^{16c} and Occ_{Li}^{8a} is calculated by taking the minimum R_{wp} from all possible 16c In and Sn occupancies. **(d)** The minimum R_{wp} value as a function of $Occ_{Li}^{16c}/Occ_{Li}^{8a}$.

Supplementary Note 5: Rietveld refinement of TOF-NPD for o-LISO

The Rietveld refinement of TOF-NPD data for o-LISO was performed using GSAS-II software. To simplify the refinement, we assumed that the o-DRX and s-phase have the same composition, which is consistent with TEM-EDS elemental mapping results indicating uniform distributions of In, Sn, and O within a particle (Fig. S12). The refinement was constrained by the composition determined from elemental analysis ($Li_{14.883}In_{8.996}SnO_{22.936}$). For the o-DRX phase, we used a typical DRX structure as the initial structure model and refined the Li occupancies at the Oct (4a) and Tet (8c) sites. Then the o-DRX structure was fixed when refining the s-phase structure. For the s-phase, we used the *Fd-3m* framework and assumed that 8b site is not occupied to avoid high-energy configurations involving Li face-sharing with high-valent In or Sn, and In/Sn do not occupy the Tet sites based on synchrotron XRD refinement (Fig. S1). Thus, the independent occupancy state variables that need to be refined for the s-phase are Occ_{Li}^{8a}, Occ_{Li}^{16c}, Occ_{In}^{16c}, and Occ_{Sn}^{16c}. A four-dimensional high throughput grid search on these site occupancies was then performed to find the s-phase structure with best fitting. As shown in Fig. S11, the 16c site in s-phase tends to be fully occupied by Li, and the Li Tet (8a) site occupancy is about 0.4. After the high throughput grid search, a manual refinement was further done to get the finally refined o-DRX and s-phase structures. Note that for s-phase, we also tried other structure frameworks (e.g., layer-like) but the fitting result is much worse than the spinel-like framework, so here we only show the refinement results based on spinel-like framework.

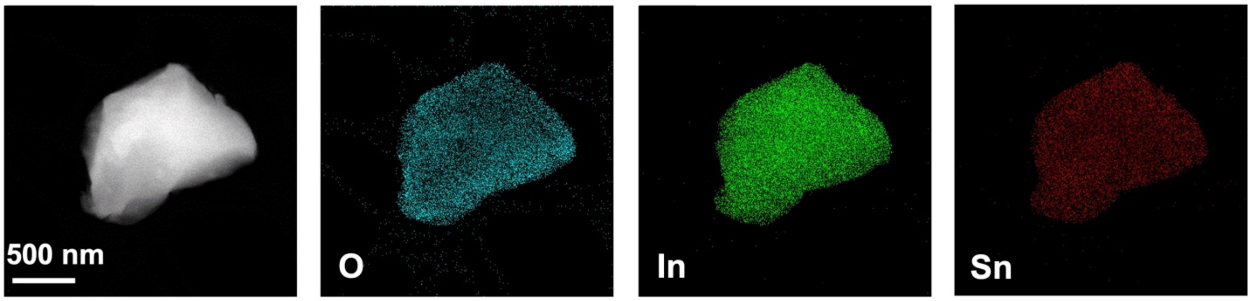

Figure S12. HAADF image of o-LISO particle and corresponding EDS mapping of In, Sn, and O elements.

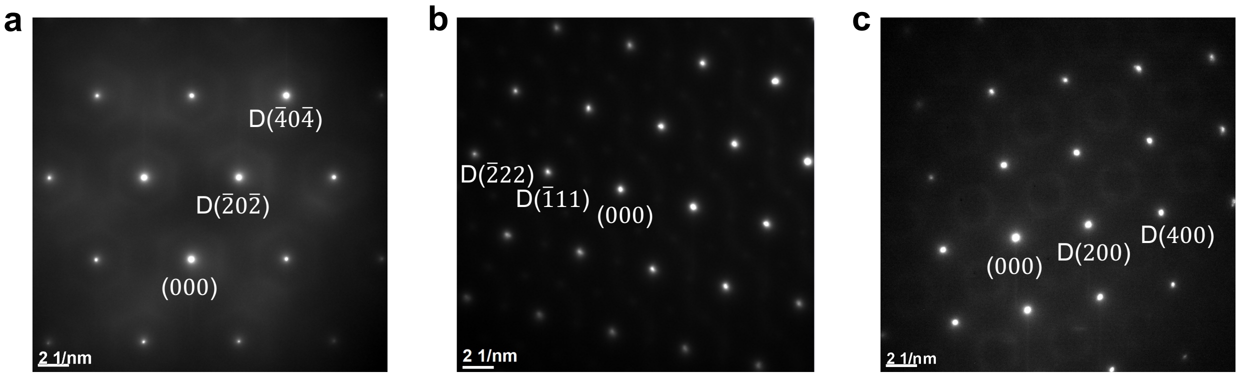

Figure S13. TEM electron diffraction pattern collected on ns-LISO particle along the zone axis of **(a)** [111], **(b)** [110], **(c)** [100].

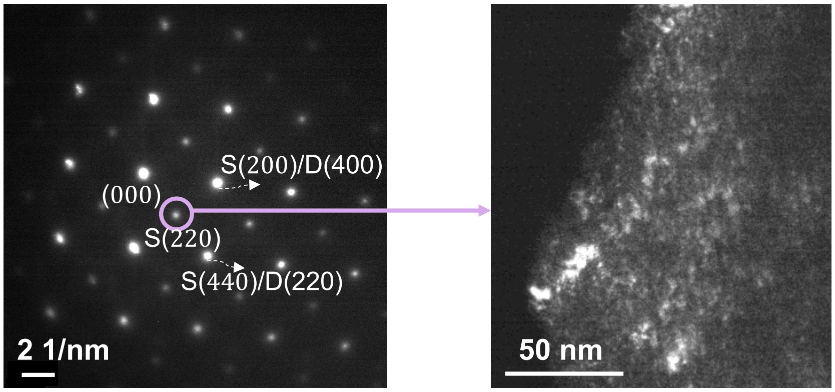

Figure S14. TEM electron diffraction pattern collected on o-LISO particle along the zone axis of [100] (left) and dark-field diffraction contrast imaging of o-LISO single particle with the selected s-phase (220) lattice plane (right). The indexed planes marked with “S” refer to s-phase, and those marked with “D” refer to the o-DRX phase.

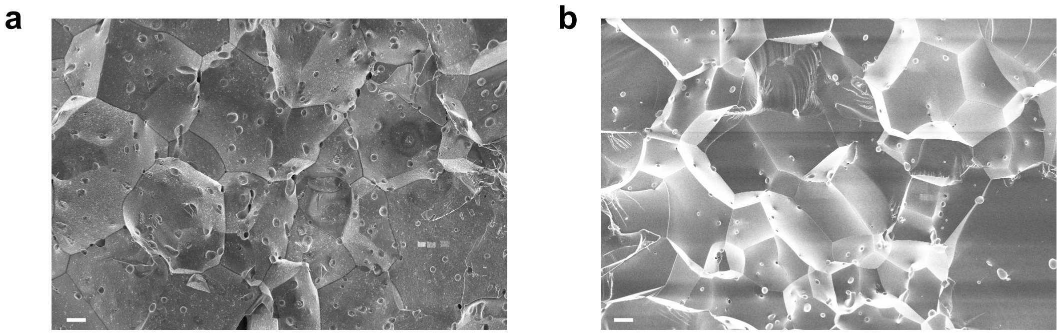

Figure S15. SEM cross-sectional images of the sintered pellets for **(a)** o-LISO and **(b)** ns-LISO. The scale bar is 10 μm.

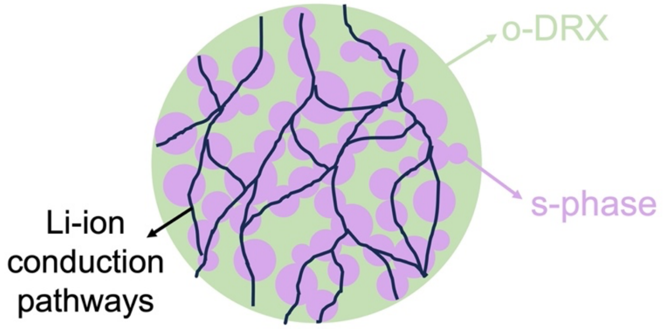

Figure S16. A schematic of Li-ion conduction pathways in o-LISO sample.

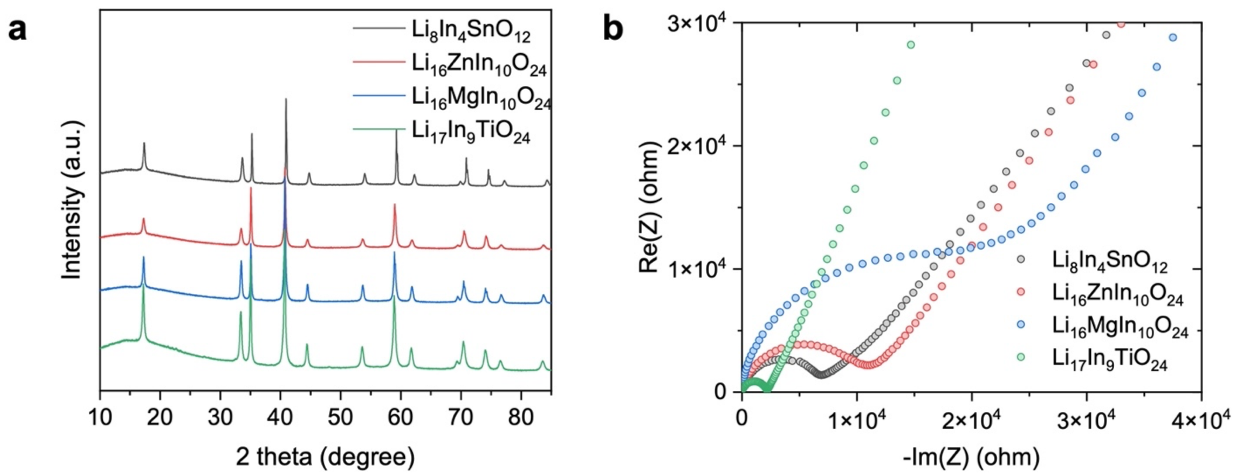

Figure S17. (a) XRD patterns of $Li_8In_4SnO_{12}$ (LISO8), $Li_{16}ZnIn_{10}O_{24}$ (LZIO16), $Li_{16}MgIn_{10}O_{24}$ (LMIO16), and $Li_{17}In_9TiO_{24}$ (LITO17) synthesized by the same solid-state method as o-LISO. **(b)** Nyquist plots of LISO8, LZIO16, LMIO16, and LITO17 from EIS measurements at RT.

Supplementary Note 6: Experimental study of ORX compounds besides o-LISO

As a demonstration of robustness of ORX compounds to chemical and compositional variations, we synthesized a series of ORX compounds by using the same solid-state method as o-LISO: $Li_8In_4SnO_{12}$ (LISO8), $Li_{16}ZnIn_{10}O_{24}$ (LZIO16), $Li_{16}MgIn_{10}O_{24}$ (LMIO16), and $Li_{17}In_9TiO_{24}$ (LITO17). As shown in Fig. S17a, s-phase with DRX form in all these compounds with Li over-stoichiometry, similar to o-LISO. The EIS measured Li-ion conductivities at RT are 4.90×10^{-5} S cm^{-1} for LISO8, 2.64×10^{-5} S cm^{-1} for LZIO16, 1.39×10^{-5} S cm^{-1} for LMIO16, and 1.41×10^{-4} S cm^{-1} for LITO17 (Fig. S17b). The Li-ion conductivities in these ORX compounds are all significantly improved than those in the corresponding stoichiometric rocksalt compounds, which are too low to be detected.

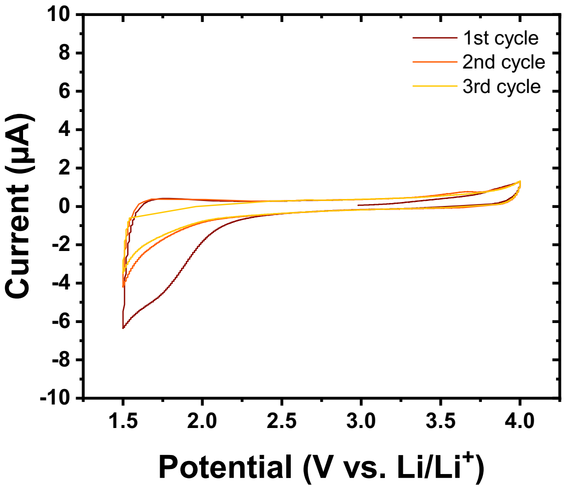

Figure S18. Cyclic voltammetry of Li/liquid electrolyte/o-LISO-C cell. The scan rate is 0.1 mV/s.

Supplementary Note 7: Electrochemical and chemical stabilities of o-LISO.

To probe the electrochemical stability of o-LISO, we performed cyclic voltammetry (CV) tests with a cell configuration: Li/liquid electrolyte/o-LISO-carbon. The o-LISO-carbon composite was used as an electrode is to ensure a large contact area between o-LISO and electronically conductive additives, which is important to measure the intrinsic electrochemical stability window of solid electrolytes[18]. The liquid electrolyte configuration was used instead of solid-state cell because of the poor stability between o-LISO and Li metal. The result of the CV test is shown in Figure S18, and indicates good electrochemical stability within ~ 2.3−4 V. The reductive stability of o-LISO is poor, which is likely due to the presence of In^{3+} which can be easily reduced by Li metal. We envision that this problem may be solved by replacing In^{3+} with more reductively stable cations, or by the use of boundary layers. The oxidative stability of o-LISO is similar to the well-known Li-garnet $Li_7La_3Zr_2O_{12}$ (LLZO), whose apparent oxidation starts at about 4.0 V[18]. This suggests that o-LISO has good oxidative stability, similar to other oxide Li superionic conductors.

We calculated the chemical reaction energy of o-LISO with three well-known cathodes, $LiCoO_2$ (LCO), $Li(NiMnCo)_{1/3}O_2$ (NMC111), $LiFePO_4$ (LFP) in their lithiated and (partially) delithiated states using the methodology by Richards et al[19]. The predicted reactions and their reaction energies E_{rxn} are summarized in Table S4. A more negative E_{rxn} indicates a higher reactivity. These results indicate that o-LISO appears fully stable with LCO and NMC111, while it is slightly unstable with their delithiated states ($E_{rxn} \sim -0.1$ eV/atom). Typically, such small negative E_{rxn} do not lead to reactivity due to kinetic limitations. We did similar calculations for LLZO and $Li_7P_3S_{11}$ (LPS) and the results are listed in Table S4. We find that o-LISO has slightly better stability with NMC111 than LLZO, while exhibits similar stability with LCO and LFP to LLZO. The cathode compatibility of o-LISO is significantly better than for sulfide-type solid electrolytes (e.g., LPS).

Table S1. The deconvoluted chemical shift, width, fraction, T_1 and T_2 for two sites from variable-temperature ^{6}Li MAS ssNMR spectra of o-LISO.

Calibrated T	Site 1 (tetrahedral)					Site 2 (octahedral)				
	Chemical Shift (ppm)	Width (ppm)	Integrated intensity fraction (%)	T1(ms)	T2(us)	Chemical Shift (ppm)	Width (ppm)	Integrated intensity fraction (%)	T1(ms)	T2(ms)
386	5.87	6.75	54.02	5.2	800	0.08	1.93	45.98	149	11.2
376	6.18	6.8	52.03	5.1	782	0.08	1.93	47.97	162.5	10.9
366	6.44	7.44	52.49	4.7	786	0.09	1.92	47.51	135.8	11.1
356	6.7	6.84	46.77	5.4	745	0.05	1.92	53.23	195.6	10.9
346	6.94	7.48	47.8	5.3	692	0.07	1.92	52.2	190	11.2
336	7.28	7.73	45.22	4.9	685	0.06	1.91	54.78	196.7	12
326	7.5	8.26	43.29	5.6	625	0.05	1.91	56.71	276.9	11.3
316	7.78	9.45	43.02	5.7	586	0.07	1.91	56.98	306.7	11.3
306	7.96	9.63	39.33	6.1	505	0.05	1.91	60.67	349.1	10.4
296	8.27	10.58	36.93	6.4	463	0.04	1.92	63.07	376.7	10.7
286	8.33	12.6	36.32	6.8	415	0.05	1.93	63.68	419.7	10.1
276	8.26	14.08	34.98	7.9	398	0.06	1.95	65.02	457.1	10.4
266	7.88	15.76	32.9	8.4	344	0.04	1.96	67.1	509	9.8
256	7.88	16.62	30.4	8.9	336	0.02	1.98	69.6	525.7	9.9
246	7.03	18.7	27.92	10.1	319	0.01	2	72.08	556.1	9.3
226	6.11	21.11	17.97	13.3	285	0	2.02	82.03	664.8	8.6

Note: The fraction of site 1 may be underestimated because of the low MAS spinning rate and very short T_2.

Table S2. The measured metal atomic ratios of o-LISO and ns-LISO from elemental analysis.

Samples	Measured ratio (Li: In: Sn)	Ratio in the initial precursors (Li: In: Sn)
o-LISO	14.9: 9.0: 1	17: 9: 1
ns-LISO	12.7: 9.1: 1	

Note that the measured Li contents in both samples are lower than the initial composition because of the Li-loss during high-temperature heat treatment. But still o-LISO is over-stoichiometric and the Li content in o-LISO is much higher than ns-LISO.

Table S3. Crystallographic data of o-DRX and s-phase in o-LISO as obtained from Rietveld refinement of TOF-NPD data.

o-DRX (64.5%): a = 4.4225(1) Å (Fm-$3m$)						
Atom	Site	x	y	z	U_{iso} (Å^2)	Occupancy
Li1	4a	0	0	0	0.115(3)	0.536(9)
Li2	8c	0.25	0.25	0.25	0.115(3)	0.058(7)
In1	4a	0	0	0	0.0022(2)	0.3913
Sn1	4a	0	0	0	0.0022(2)	0.0435
O1	4b	0.5	0.5	0.5	0.0048(1)	1
s-phase (35.5%): a = 8.8517(4) Å (Fd-$3m$)						
Atom	Site	x	y	z	U_{iso} (Å^2)	Occupancy
Li1	16d	0.5	0.5	0.5	0.0131(11)	0.1044
Li2	16c	0	0	0	0.0131(11)	1
Li3	8a	0.125	0.125	0.125	0.0131(11)	0.4
In1	16d	0.5	0.5	0.5	0.0045(5)	0.7826
Sn1	16d	0.5	0.5	0.5	0.0045(5)	0.0869
O1	32e	0.2581(1)	0.2581(1)	0.2581(1)	0.0033(2)	1
R_{wp} = 7.41%, GOF = 3.95						

Note that since the Li site occupancies in s-phase were obtained by high throughput grid search, their uncertainties are not provided.

Table S4. The summary of calculated interfacial chemical reaction energies and the reaction equations between solid electrolytes (o-LISO, $Li_7La_3Zr_2O_{12}$, $Li_7P_3S_{11}$) and known cathodes ($LiCoO_2$, $Li(NiMnCo)_{1/3}O_2$, $LiFePO_4$) with both lithiated and delithiated states.

Solid electrolyte	Cathode	E_{rxn} (eV/atom)	Reaction (Normalized to reflect molar fraction)
o-LISO	$LiCoO_2$	0	/
	CoO_2	−0.106	0.11 $Li_{17}In_9SnO_{24}$ + 0.89 CoO_2 → 0.89 Li_2CoO_3 + 0.11 SnO_2 + 0.47 In_2O_3
	$Li(NiMnCo)_{1/3}O_2$	0	/
	$(NiMnCo)_{1/3}O_2$	−0.093	0.21 $Li_{17}In_9SnO_{24}$ + 0.79 $MnCoNiO_6$ → 0.03 LiO_8 + 0.39 $Li(NiO_2)_2$ + 0.79 Li_2CoO_3 + 0.79 Li_2MnO_3 + 0.21 SnO_2 + 0.95 In_2O_3
	$LiFePO_4$	−0.088	0.11 $Li_{17}In_9SnO_{24}$ + 0.90 $LiFePO_4$ → 0.03 Fe_3O_4 + 0.04 $FeSnO_3$ + 0.76 FeO + 0.06 $In_{15}SnO_{24}$ + 0.90 Li_3PO_4
	$FePO_4$	−0.170	0.15 $Li_{17}In_9SnO_{24}$ + 0.85 $FePO_4$ → 0.43 Fe_2O_3 + 0.68 In_2O_3 + 0.15 SnO_2 + 0.85 Li_3PO_4
$Li_7La_3Zr_2O_{12}$	$LiCoO_2$	0	/
	CoO_2	−0.101	0.22 $Li_7La_3Zr_2O_{12}$ + 0.78 CoO_2 → 0.22 $La_2Zr_2O_7$ + 0.78 Li_2CoO_3 + 0.11 La_2O_3
	$Li(NiMnCo)_{1/3}O_2$	−0.091	0.40 $Li_7La_3Zr_2O_{12}$ + 0.60 $Li_3MnCoNiO_6$ → 0.60 $LiNiO_2$ + 0.40 $Li_6Zr_2O_7$ + 0.60 La_2MnCoO_6 + 0.80 Li_2O
	$(NiMnCo)_{1/3}O_2$	−0.189	0.40 $Li_7La_3Zr_2O_{12}$ + 0.60 $MnCoNiO_6$ → 0.08 LiO_8 + 0.76 Li_2ZrO_3 + 0.60 Li_2NiO_3 + 0.60 La_2MnCoO_6 + 0.04 ZrO_2
	$LiFePO_4$	−0.091	0.22 $Li_7La_3Zr_2O_{12}$ + 0.78 $LiFePO_4$ → 0.22 $La_2Zr_2O_7$ + 0.22 $LaFeO_3$ + 0.44 FeO + 0.78 Li_3PO_4 + 0.11 Fe
	$FePO_4$	−0.183	0.16 $Li_7La_3Zr_2O_{12}$ + 0.84 $FePO_4$ → 0.37 Li_3PO_4 + 0.47 $LaPO_4$ + 0.42 Fe_2O_3 + 0.32 ZrO_2
$Li_7P_3S_{11}$	$LiCoO_2$	−0.456	0.14 $Li_7P_3S_{11}$ + 0.86 $LiCoO_2$ → 0.21 CoS_2 + 0.21 Co_3S_4 + 0.29 Li_2S + 0.43 Li_3PO_4
	CoO_2	−0.761	0.09 $Li_7P_3S_{11}$ + 0.91 CoO_2 → 0.27 $LiCoPO_4$ + 0.01 Co_9S_8 + 0.18 Li_2SO_4 + 0.19 Co_3S_4
	$Li(NiMnCo)_{1/3}O_2$	−0.415	0.33 $Li_7P_3S_{11}$ + 0.67 $Li_3MnCoNiO_6$ → 0.22 $Co(NiS_2)_2$ + 0.22 $Li(MnS_2)_2$ + 0.22 Co_2NiS_4 + Li_3PO_4 + 0.22 MnS_2 + 0.56 Li_2S
	$(NiMnCo)_{1/3}O_2$	−0.756	0.23 $Li_7P_3S_{11}$ + 0.77 $MnCoNiO_6$ → 0.24 $Co(NiS_2)_2$ + 0.68 $LiMnPO_4$ + 0.01 Ni_3S_2 + 0.46 Li_2SO_4 + 0.09 MnO + 0.27 Co_2NiS_4
	$LiFePO_4$	−0.129	0.14 $Li_7P_3S_{11}$ + 0.87 $LiFePO_4$ → 0.39 $Li_4P_2O_7$ + 0.25 FePS + 0.24 $LiPO_3$ + 0.62 FeS_2
	$FePO_4$	−0.224	0.16 $Li_7P_3S_{11}$ + 0.84 $FePO_4$ → 0.02 P_4S_7 + 0.10 FePS + 1.12 $LiPO_3$ + 0.74 FeS_2

References:

1 Shi, X. *et al.* Fast Li-ion Conductor of Li_3HoBr_6 for Stable All-Solid-State Lithium–Sulfur Battery. *Nano Letters*, doi:10.1021/acs.nanolett.1c03573 (2021).

2 Hodge, I. M., Ingram, M. D. & West, A. R. Impedance and modulus spectroscopy of polycrystalline solid electrolytes. *Journal of Electroanalytical Chemistry and Interfacial Electrochemistry* **74**, 125-143, doi:https://doi.org/10.1016/S0022-0728(76)80229-X (1976).

3 Irvine, J. T. S., Sinclair, D. C. & West, A. R. Electroceramics: Characterization by Impedance Spectroscopy. *Advanced Materials* **2**, 132-138, doi:https://doi.org/10.1002/adma.19900020304 (1990).

4 Aono, H., Imanaka, N. & Adachi, G.-y. High Li+ conducting ceramics. *Accounts of chemical research* **27**, 265-270 (1994).

5 Fu, J. Fast Li+ ion conducting glass-ceramics in the system $Li_2O–Al_2O_3–GeO_2–P_2O_5$. *Solid State Ionics* **104**, 191-194 (1997).

6 Illbeigi, M., Fazlali, A., Kazazi, M. & Mohammadi, A. H. Effect of simultaneous addition of aluminum and chromium on the lithium ionic conductivity of $LiGe_2(PO_4)_3$ NASICON-type glass–ceramics. *Solid State Ionics* **289**, 180-187 (2016).

7 Aono, H., Sugimoto, E., Sadaoka, Y., Imanaka, N. & Adachi, G.-y. Electrical Properties and Sinterability for Lithium Germanium Phosphate $Li_{1+x}M_xGe_{2-x}(PO_4)_3$, M= Al, Cr, Ga, Fe, Sc, and In Systems. *Bulletin of the Chemical Society of Japan* **65**, 2200-2204 (1992).

8 Xiong, L. *et al.* LiF assisted synthesis of $LiTi_2(PO_4)_3$ solid electrolyte with enhanced ionic conductivity. *Solid State Ionics* **309**, 22-26 (2017).

9 Zhang, Z. *et al.* New horizons for inorganic solid state ion conductors. *Energy & Environmental Science* **11**, 1945-1976, doi:10.1039/C8EE01053F (2018).

10 Ibarra, J. *et al.* Influence of composition on the structure and conductivity of the fast ionic conductors $La_{2/3-x}Li_{3x}TiO_3$ ($0.03 \leqslant x \leqslant 0.167$). *Solid State Ionics* **134**, 219-228 (2000).

11 Mazumdar, D., Bose, D. & Mukherjee, M. Transport and dielectric properties of lisicon. *Solid state ionics* **14**, 143-147 (1984).

12 Zhao, G. *et al.* Extending the Frontiers of Lithium-Ion Conducting Oxides: Development of Multicomponent Materials with γ-Li_3PO_4-Type Structures. *Chemistry of Materials* **34**, 3948-3959, doi:10.1021/acs.chemmater.1c04335 (2022).

13 Zhao, G. *et al.* High lithium ionic conductivity of γ-Li_3PO_4-type solid electrolytes in Li_4GeO_4– Li_4SiO_4–Li_3VO_4 quasi-ternary system. *Journal of Solid State Chemistry* **292**, 121651 (2020).

14 Kuwano, J. & West, A. New Li+ ion conductors in the system, Li_4GeO_4-Li_3VO_4. *Materials Research Bulletin* **15**, 1661-1667 (1980).

15 Wang, Q. *et al.* A new lithium‐ion conductor $LiTaSiO_5$: theoretical prediction, materials synthesis, and ionic conductivity. *Advanced Functional Materials* **29**, 1904232 (2019).

16 Kim, R. *et al.* Computational Design and Experimental Synthesis of Air-Stable Solid-State Ionic Conductors with High Conductivity. *Chemistry of Materials* **33**, 6909-6917, doi:10.1021/acs.chemmater.1c01837 (2021).
17 Jones, M. A. *et al.* Short-range ordering in a battery electrode, the 'cation-disordered' rocksalt Li1.25Nb0.25Mn0.5O2. *Chemical Communications* **55**, 9027-9030, doi:10.1039/C9CC04250D (2019).
18 Han, F., Zhu, Y., He, X., Mo, Y. & Wang, C. Electrochemical Stability of Li10GeP2S12 and Li7La3Zr2O12 Solid Electrolytes. *Advanced Energy Materials* **6**, 1501590, doi:https://doi.org/10.1002/aenm.201501590 (2016).
19 Richards, W. D., Miara, L. J., Wang, Y., Kim, J. C. & Ceder, G. Interface Stability in Solid-State Batteries. *Chemistry of Materials* **28**, 266-273, doi:10.1021/acs.chemmater.5b04082 (2016).
